# Starch mediates and cements densely magnetite-coating of talc, giving an efficient nano-catalyst for three-component synthesis of imidazo[1,2-*c*]quinazolines

**DOI:** 10.1038/s41598-023-51123-y

**Published:** 2024-01-05

**Authors:** Hedyeh Hosseinzadeh, Kurosh Rad-Moghadam, Morteza Mehrdad, Somayeh Rouhi

**Affiliations:** Chemistry Department, University of Guilan, Rasht, 41335-1914 Iran

**Keywords:** Chemistry, Nanoscience and technology

## Abstract

Hot-water-soluble starch (HWSS) was used as a powerful cementing material to produce nano-size conglomerates of talc and magnetite nanoparticles. Coordination of HWSS hydroxyl groups to iron atoms at surface of magnetite leads to grafting and encapsulation of its nanoparticles. The resulting nano-complex showed a higher loading capacity on talc than pristine magnetite nanoparticles. Only a minute amount of HWSS was detected in the fabricated nano-composite Talc\HWSS@Fe_3_O_4_. XPS study suggests a considerable interaction between HWSS and Fe_3_O_4_ nanoparticles, upon which some of the Fe^+3^ atoms on surface of Fe_3_O_4_ are reduced into Fe^+2^ atoms. ATR FT-IR spectra of the nano-composite revealed significant delamination of talc sheets on interaction with HWSS-coated Fe_3_O_4_ nanoparticles. The nano-composite displayed an efficient catalytic activity in the synthesis of new imidazo[1,2-*c*]quinazoline derivatives via Grobke–Blackburn–Bienaymé three-component reaction of 4-aminoquinazoline, arylaldehydes and isocyanide. The efficiency of the method was exemplified by synthesizing 7 new products in fairly high yields (68–83%) within short reaction times (24–30 min) using a catalytic amount of the catalyst under solvent-free condition at 120 °C. Clean and fast synthesis of the products and convenient separation of the robust nano-catalyst are the prominent advantages of the present method. The nano-catalyst was properly characterized.

## Introduction

Catalysis is the central reaction of most chemical transformations and is the key process to economizing numerous chemical productions^1^. Hence, it is not surprising that huge amounts of catalysts are annually employed around the world in chemical industries and because of their limited durability should be ultimately discarded to environment as industrial wastes. Increasing pollution of the environment and its harmful effects on human health has urged the chemical community to develop new methods based on using biocompatible catalysts and green chemical procedures^2^. According to the principles of green chemistry, design and use of environmentally benign catalysts with the advantages of low preparation cost, high activity, efficient recovery, and good recyclability, is more demanded^3^. In this view, heterogeneous catalysts have come to the fore as easily separable and recyclable materials in most recent research and developments^4–8^. However, heterogeneous catalysts have small surfaces to contact with reactants, so are usually less effective catalysts than the homogeneous types and thus should be used in larger amounts. This disadvantage can be considerably surmounted by reducing the size of these catalysts to nano-dimensions. Solid catalysts would acquire a great surface per mass ratio and surface energy as their size is reduced to nano-dimensions. Moreover, the outermost metal atoms of the nano-catalysts are not capable to complete their valance capacity all by formation of covalent bonds but form some dangling bonds liable to displacement, so are more reactive than the inner atoms and the atoms on surface of bulky particles. Although of these prominent advantageous features, nanoparticles require special techniques such as fine filtration or ultracentrifugation to be separated from reaction mixtures after use. An intriguing strategy to facilitate the separation of nanoparticles is to equip them with a magnetic core and perform their isolation by the so-called “magnetic separation” method using a permanent magnet^9–12^. Magnetite has been by far the most attractive magnetic material widely used for fabrication of diverse magnetic nano-composites^13^. It is a low-toxic, low-cost, chemically stable, biocompatible and readily available mixed iron oxide^14^. The magnetic regime of magnetite significantly depends on size of its particles. By reducing the size of bulky magnetite particles to nano-scales they turn from ferrimagnetic into superparamagnetic particles and this change becomes more evident below a critical dimension close to the size of a single magnetic zone^15^. Superparamagnetic particles have no net magnetic moments in the absence of an external magnetic field, hence can be dispersed homogeneously in liquids, since no aggregating magnetic force they exert to each other. On the other hand, in the field of an applied external magnet they acquire sufficiently strong magnetic moments to be separated from mixtures by using a permanent magnet. Recently, we showed that magnetite has a great tendency to coordinate with hot-water-soluble starch (HWSS), leading to deaggregation and encapsulation of its nanoparticles by HWSS^11^. Other researchers reported their experiences on significant adhesion of starch to talc^16–18^. Taking these facts into consideration, we aimed at using HWSS as a cementing component to fabricate a bio-derived nano-composite from magnetite and talc. Chemical formula of talc is known as Mg_3_Si_4_O_10_(OH)_2_ and it has a layer-lattice structure consisting of a magnesium hydroxide layer (MgO-H_2_O) sandwiched in between two silicate (Si_2_O_5_) layers. With this structure, talc features a heterogeneous surface comprising of a hydrophobic silica face and a hydrophilic edge decorated by Mg-OH and Si–OH functions. Therefore, it is not capable to load significant amounts of magnetite nanoparticles on the exterior surface of its silica layers. Transmission electron microscopy (TEM) images showed that magnetite nanoparticles prefer to attach to edges of talc flakes and additional experiments revealed that the resulting nano-composite is not stable for a long time in aqueous medium^19^. Nevertheless, the nano-composite still proved to be useful for adsorptive remediation of hazardous pollutants from waste waters^20,21^. Laskowski reported that metal hydroxides already adsorbed on surface of silica do enhance its capacity for adsorption of starch^22^. In this background and in continuation of our previous works on production of catalysts^23,24^ especially from HWSS-coated magnetite nanoparticles^5,11,25^, we describe here an increased loading of magnetite nanoparticles on talc. The as-prepared nano-composite displayed an efficient catalytic activity in the Grobke–Blackburn–Bienaymé synthesis of imidazo[1,2-*c*]quinazolines from one-pot reaction of arylaldehyde, isocyanide, and 4-aminoquinazoline. Quinazolines have long been the subject of numerous studies, due to their diverse pharmaceutical and biological activities^26–29^. As a result, many efforts have been devoted to synthesis and pharmaceutical screening of quinazoline derivatives that led in part to the emergence of imidazo[1,2-*c*]quinazolines as recurrent themes in drug developments. Compounds consisting of imidazo[1,2-*c*]quinazoline framework display a wide range of biological and medicinal properties such as antimicrobial^30^, lipid peroxidation inhibitory^31^, and PI3-kinase inhibitory activities^32^. These valuable properties combined with the utility of imidazo[1,2-*c*]quinazolines in production of metal ion-sensors^33,34^, electroluminescent organic component of OLEDs^35^, and molecular rotors^36^ account for the vast efforts have been paid to development of diverse routes to their synthesis. Despite of significant differences between the reported synthetic methods, they can be classified into two main groups according to their key reactions through which either the imidazole^37–39^ or the pyrimidine ring^40–48^ is annulated. Moreover, fascinating advances have been achieved in one-step construction of the both rings^49^ and in using simple starting materials^50^. Of particular importance in this context are the multicomponent synthetic routes through which an imidazole ring is annulated onto a readily available quinazoline framework^51^. Since these reactions are really shortcuts for expanding the known library of imidazo[1,2-*c*]quinazolines and to perform diversity oriented syntheses. A literature survey has disclosed only one such route to imidazo[1,2-*c*]quinazolines^39^ along with scarce cases for synthesis of analogous compounds^52,53^. However, despite of their own merits, these synthetic reactions are too long, perhaps due to unsuitability of their catalytic conditions. These synthetic routes are commonly featured by the Grobke–Blackburn–Bienaymé reaction on a fused aminopyrimidine ring, so can be considered as the extensions of this reaction, which was initially developed by using 2-aminopyridine and other aminoazines as substrates. A majority of the many Brønsted and Lewis acidic catalysts, which have been introduced for development of this reaction, are homogeneous materials hardly separable from the reaction mixtures after use^39,52–54^. On the other hand, some of the previously applied heterogeneous catalysts are expensive^55^ and difficultly available nano-materials^56^, take long times or give low yields of the products^57^, need be used in virtually large amounts and require the special technique of microwave irradiation to show a sufficient efficiency^58^. Therefore, challenges still remain in designing green and easily separable catalysts, which are also capable to delivering the products in high yields within short reaction times. Here, we introduce Talc\HWSS@Fe_3_O_4_, as a magnetically retrievable nano-catalyst, for three-component synthesis of some new imidazo[1,2-*c*]quinazolines within short reaction times under a solvent-free condition.

## Results and discussion

**Figure 1:**
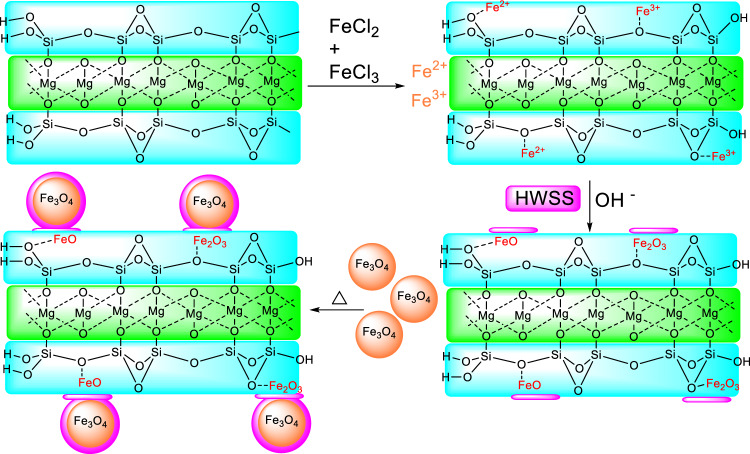
The schematic procedure for preparation of the nano-composite Talc\HWSS@Fe_3_O_4_.

A two-step protocol was developed for fabrication of the nano-composite, Talc\HWSS@Fe_3_O_4_. As outlined in Fig. 1, Fe_3_O_4_ NPs were prepared by co-precipitation of Fe^III^ and Fe^II^ oxides in an alkaline suspension of talc. Addition of an aqueous solution of starch to this suspension followed by refluxing the combined fluid gave the nano-composite of talc and magnetite NPs, in which the components were adhered by thin films of starch (Fig. 1).

**Figure 2:**
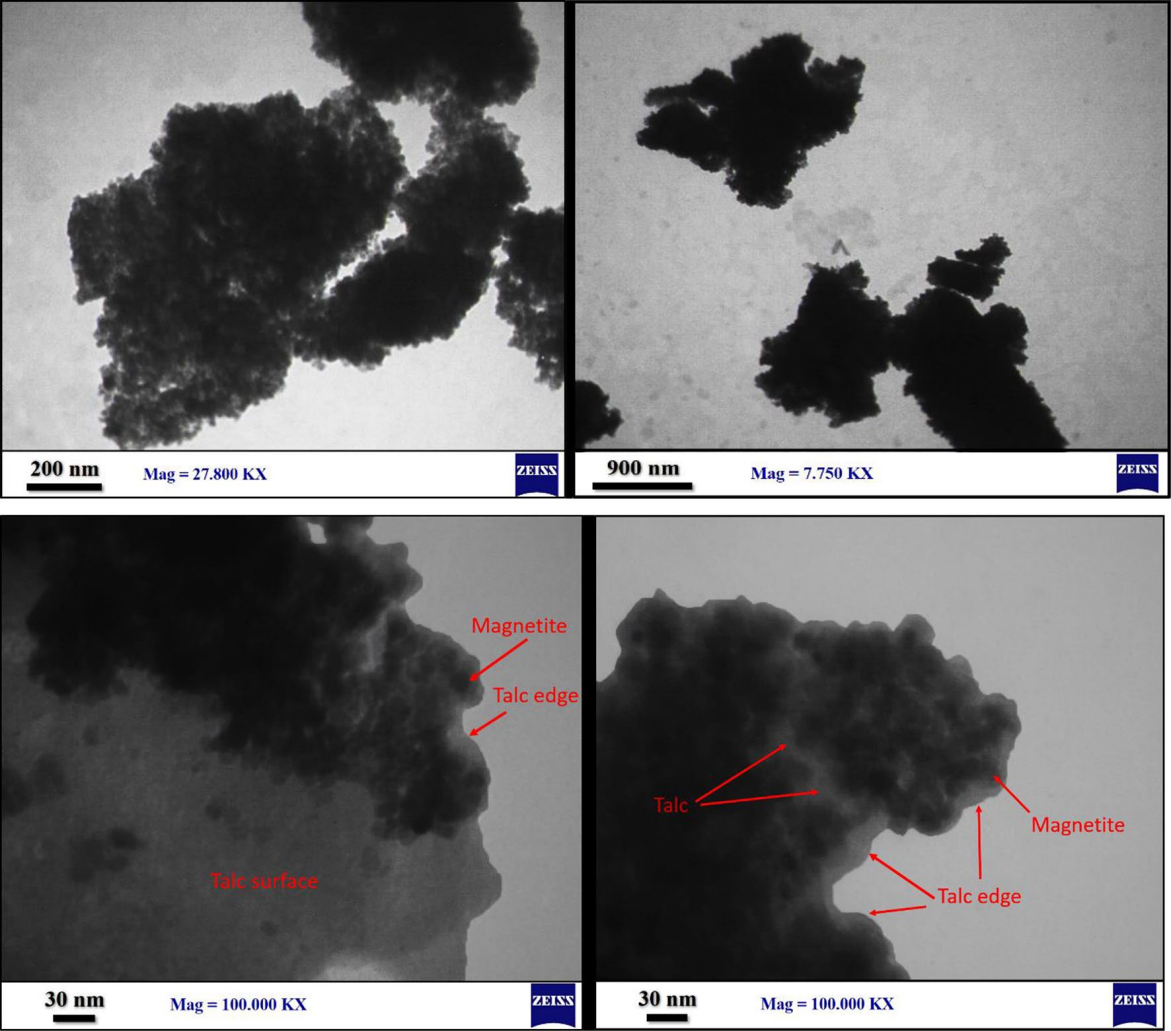
TEM images of Talc\HWSS@Fe_3_O_4_ prepared via in situ formation of Fe_3_O_4_ NPs in a suspension of talc and an aqueous starch solution.

Because of small size of the magnetite NPs, a majority of the iron atoms at surface of these NPs are connected with less bridging bonds to other iron atoms of the lattice, so have a great tendency to complete their valence by coordination with the hydroxyl groups of HWSS. Parts of these coordination interactions may lead to formation of covalent C-O-Fe bonds and thus chemisorption of HWSS molecules onto magnetite NPs. Adhesion of HWSS to magnetite NPs is certainly reinforced effectively by physisorption involving H-bonding and dispersive attraction forces. On the other hand, adsorption of HWSS onto talc is not so extensive, since it limits to just physisorption forces, including H-bonding and coordination of its hydroxyl groups with Mg_3_(OH)_2_ layer of talc at edges and its hydrophobic adsorption on the silica layers. Yet, pre-adsorption of iron ions on exterior surface of silica platelets in talc, and then their conversion at there to iron hydroxides in alkali medium is perceived to increase the adsorption of starch and HWSS@Fe_3_O_4_ NPs. TEM images of the nano-composite, containing the nominal 77 weight percent of talc (relative to the Fe_3_O_4_ content), displayed the talc flakes nearly completely coated with magnetite NPs (Fig. 2). Due to heterogeneous nature of the preparation method, there are seen scarce TEM images in which the surface of talc flakes has been partially coated by HWSS@Fe_3_O_4_ NPs. Close inspection of these images and those of the entirely coated talc sheets indicates that the edges of the sheets have been left largely uncoated by HWSS@Fe_3_O_4_ NPs. This finding can be interpreted as positively charged iron cations have less tendency to adsorb at edges and near the termini of Mg_3_(OH)_2_ layer, leading HWSS@Fe_3_O_4_ NPs to adsorb slightly far from the talc edges. Therefore, the free edges of talc in this nano-composite are available as catalytic sites to representative acid-catalyzed reactions.

**Figure 3:**
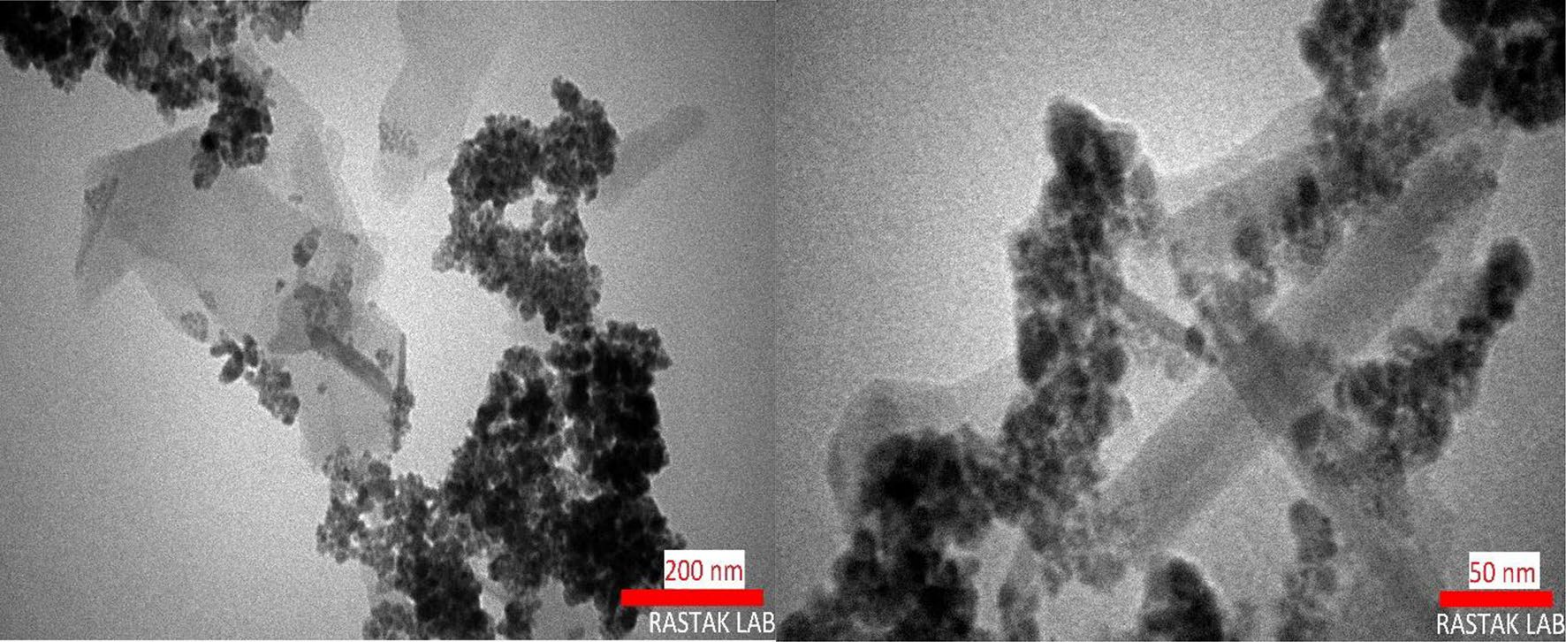
TEM images of Talc\HWSS@Fe_3_O_4_ fabricated via refluxing the pre-prepared HWSS@Fe_3_O_4_ NPs in a suspension of talc and aqueous starch solution.

In order to verify the leading role of the pre-adsorbed iron cations, coating of HWSS@Fe_3_O_4_ NPs onto talc was tested in the absence of iron cations. Figure 3 exhibits the TEM images of the Talc\HWSS@Fe_3_O_4_ nano-composite prepared by refluxing a suspension of talc and pre-prepared HWSS@Fe_3_O_4_ NPs in an ion-free aqueous solution of starch. As this figure shows, the ex situ prepared HWSS@Fe_3_O_4_ NPs have less tendency to adsorb onto talc sheets. A significant amount of these NPs aggregated and a slight amount of them were seen as attached to edges of the talc sheets.

**Figure 4:**
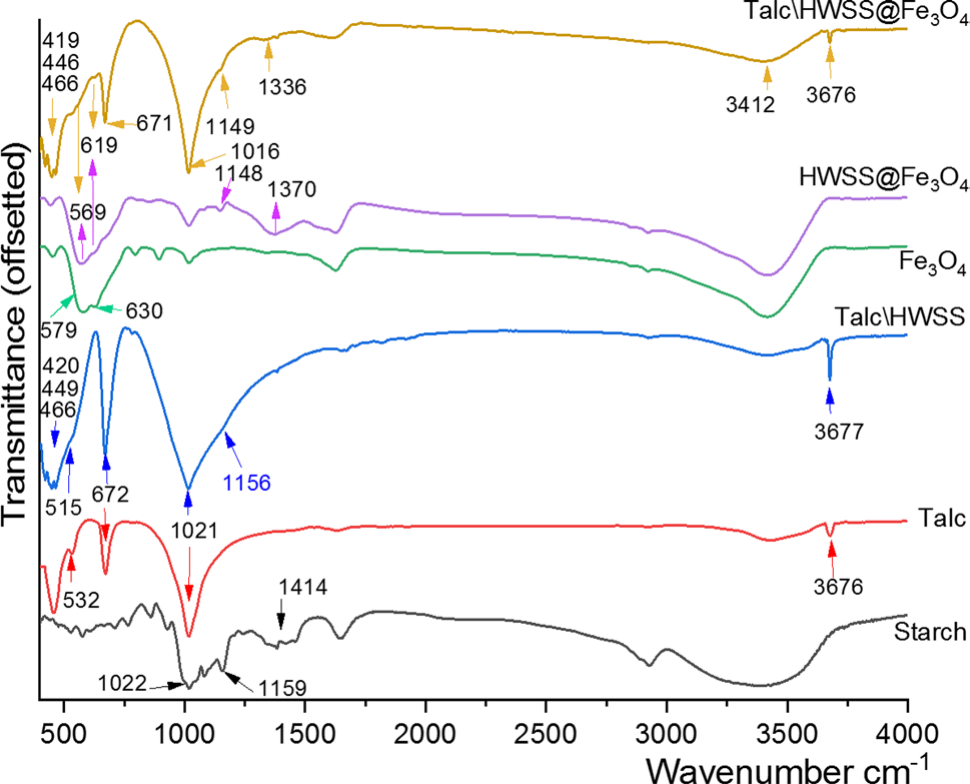
Comparative transmission IR spectra of Talc, Talc\HWSS, Fe_3_O_4_, Fe_3_O_4_@HWSS, and Talc\HWSS@Fe_3_O_4_.

Valuable insights into the structure of the nano-composite is obtained by comparing its FT-IR spectrum with those of magnetite, talc and HWSS (Fig. 4). Transmission IR spectrum of dry HWSS displays bands at 1462, 1420, 1382 and 1345 cm^−1^, which are assigned to its CH_2_ deformation, C–H bending, CH_2_ twisting and C–O–H bending vibrations, respectively^59,60^. These bands become significantly flattened in the IR spectrum of Talc\HWSS and are seen merged with the blue-drifts of the Si–O–Si stretching band of talc at 1021 cm^−1^. The effect of Fe_3_O_4_ on these HWSS bands is even more evident, as in the IR spectra of HWSS@Fe_3_O_4_ and Talc\HWSS@Fe_3_O_4_ they coalesce into a broad band with the center red-shifted from 1414 cm^−1^ to 1370 and 1336 cm^−1^ for the two magnetic composites, respectively. In the IR spectrum of Talc\HWSS, the three C-O stretching bands of HWSS at 1159, 1085 and 1022 cm^−1^ are seen as overlapped with the Si–O–Si phonon stretching band of Talc, and no appreciable shift can be detected for at least the former band. Based on these observations one may conclude that the interaction between HWSS and talc is mainly hydrophobic in nature. Since, only the C–H/CH_2_ bending and Si–O stretching vibrations rather than the C–O stretching bands have been faintly affected by the interaction between the two components. For the cases of HWSS@Fe_3_O_4_ and Talc\HWSS@Fe_3_O_4_, however, the C-O stretching bands show a slight attenuation of intensity and significant red shifts. A similar red shift is also observed for the Si–O–Si stretching band of Talc\HWSS@Fe_3_O_4_, indicating that the strength of Si–O bands of talc has been affected by the iron species adsorbed on its surface. Noteworthy, the extent of red-shift in this IR-region of the composite seems to be directly related to wavenumbers of the bands. Hence, the C–O and Si–O–Si stretching vibrations of Talc\HWSS@Fe_3_O_4_ become nearly united in a new band which peaks at 1016 cm^−1^ and is thinner than that seen for Talc\HWSS. From these findings it can be inferred that HWSS and Fe_3_O_4_ are responsible to the strongest interactions in Talc\HWSS@Fe_3_O_4_ and the iron species pre-adsorbed on the basal surface of talc behave as pins for efficient adhesion of HWSS and HWSS-coated Fe_3_O_4_ NPs to the silica facial of talc. Another fact in support of HWSS-Fe_3_O_4_ interaction comes from inspection of Fe–O stretching vibrations. In ideal spinel structure of magnetite, Fe^+2^ and Fe^+3^ atoms get located in its tetrahedral and octahedral sites, respectively. Nevertheless, this situation is not really met in natural and synthetic magnetite crystals, wherein either of the two sites are occupied by both Fe^+2^ and Fe^+3^ atoms. It is the reason Fe–O bonds in e.g. tetrahedral sites of Fe_3_O_4_ exhibit two distinctly separate stretching bands. For the pristine Fe_3_O_4_ NPs used in this work, the tetrahedral Fe–O bands appeared nearly in equal intensity at 630 and 579 cm^−1^. However, in the IR spectrum of HWSS@Fe_3_O_4_ NPs, these two bands appeared with red-shifts at 619 and 569 cm^−1^, respectively. The red-shifts are indicative of strong complexing interactions and perhaps formation of covalent bonds between HWSS and Fe_3_O_4_ NPs. Moreover, the two bands gained a different relative intensities in the doublet of tetrahedral Fe–O stretching band recorded for HWSS@Fe_3_O_4_ NPs. Presumably, a number of Fe^+3^ atoms in tetrahedral sites of Fe_3_O_4_ NPs are reduced to Fe^+2^ atoms by HWSS during production of HWSS@Fe_3_O_4_ NPs. Similar red-shifts and unequal intensities are seen for the two tetrahedral Fe–O bands of Talc\HWSS@Fe_3_O_4_. This finding indicates that the superior interaction between HWSS and Fe_3_O_4_ is not interrupted by talc. Talc itself displays characteristic bands at around 3676 cm^−1^ (Mg_3_-OH), 672 cm^−1^ (O–H deformation), 515 cm^−1^ and 449 cm^−1^ (Si–O–Mg), 466 cm^−1^ (Mg-O), and 420 cm^−1^ (Si–O)^61^. None of these bands undergo sensible shifts in both Talc\HWSS and Talc\HWSS@Fe_3_O_4_, acknowledging that talc is dispersed dominantly by hydrophobic interactions with HWSS in the two composites.

In ATR FT-IR spectrum of talc, the Si–O–Si phonon band is observed at 961 cm^−1^, which displays a certain red-shift (~ 60 cm^−1^) relative to the wavenumber of this band in transmission FT-IR spectrum of talc (see S1 in the Supporting Information (SI)). Shifts of the ATR FT-IR bands to lower wavenumbers is due to anomalous dispersion of refractive index across the absorption band. The red-shift is most prominent for the intense bands of the samples of relatively high refractive index. A similar but slightly lesser red-shift was detected for the Si–O–Si phonon band of Talc\HWSS appeared at 967 cm^−1^. The decrease of red-shift (54 cm^−1^) relative to the red-shift seen for pure talc (60 cm^−1^) can be ascribed to either a slight decrease in refractive index of sample or slight exfoliation of talc sheets by HWSS. In the case of Talc\HWSS@Fe_3_O_4_, though we were not able to measure the refractive index of this solid, it is clearly greater than that of pure talc and Talc\HWSS. This estimation is based on considering the refractive indices of magnetite (3.6 at around 970 cm^−1^), starch (1.53) and talc (1.539–1.589). Hence, the significant decrease in the red-shift (16 cm^−1^) seen for Si–O–Si phonon band of Talc\HWSS@Fe_3_O_4_ (at 1005 cm^−1^) should be resulted from exfoliation of the lamellar talc flakes by HWSS@Fe_3_O_4_ NPs. It is known that the width, intensity and wavenumber of ATR FT-IR bands are explicitly affected by the particle size of minerals. Usually, a decrease in average size of particles leads to an increase in intensity and wavenumber of ATR FT-IR bands, while the width of bands is decreased^62^. As another evidence in support of talc delamination during production of Talc\HWSS@Fe_3_O_4_ NPs, the Si–O–Si phonon band is relatively intensified and gets a smaller full-width at half-height value (FWHM = 136 cm^−1^) than the same band in ATR FT-IR spectrum of pure talc (FWHM = 188 cm^−1^). A blue-shift is also seen for Fe–O band of HWSS@Fe_3_O_4_ relative to wavenumber of this band in ATR FT-IR spectrum of Fe_3_O_4_ (see S1). This shift may be arisen due to a decrease in the refractive index of the sample and deaggregation of the Fe_3_O_4_ NPs by HWSS.

**Figure 5:**
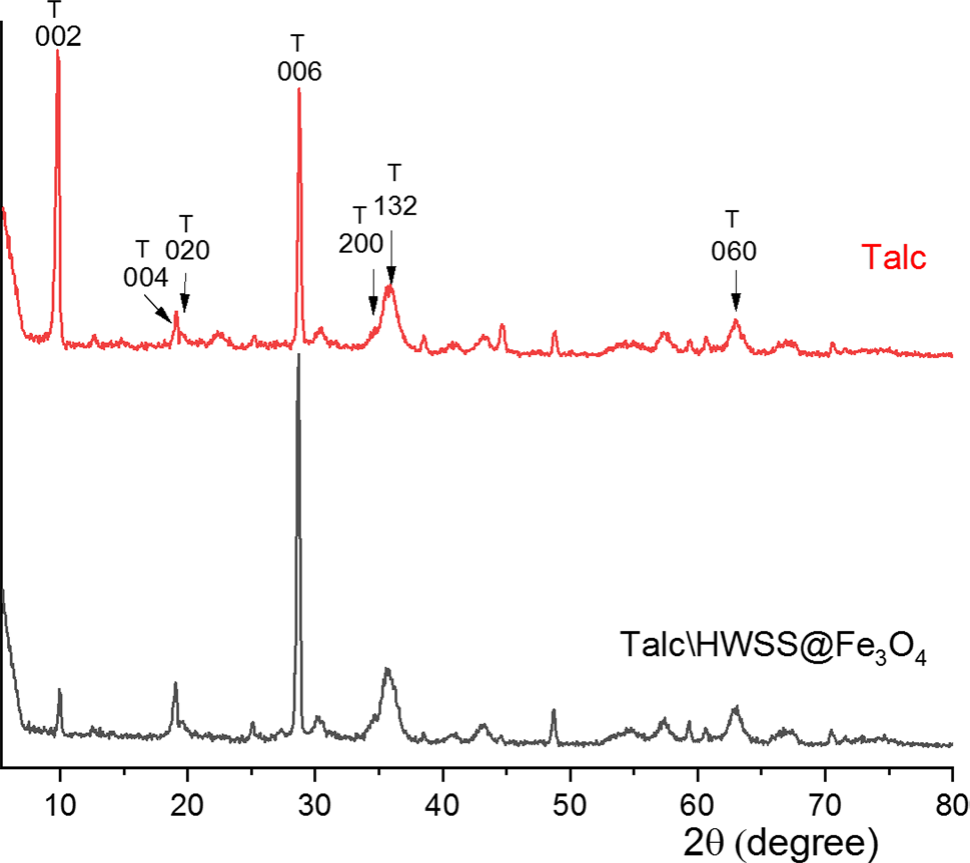
XRD patterns of talc and Talc\HWSS@Fe_3_O_4_ NPs.

Delamination of talc by HWSS@Fe_3_O_4_ NPs was verified by comparing its XRD-pattern with that of Talc\HWSS@Fe_3_O_4_ NPs (Fig. 5). The peaks seen in the XRD profile of the talc, used in this investigation, at 2θ values of 9.80°, 19.11°, 19.53°, 34.79°, 35.84°, and 63.0° are indexed to diffractions from (002), (004), (020), (006), (200), (132), and (060) planes of its crystals, respectively (ICDD card No. 19-770). Some other small peaks in the pattern are diffractions from natural impurities of talc. Almost all the characteristic peaks of talc are detected in the XRD-pattern of Talc\HWSS@Fe_3_O_4_ and almost no shifts can be discerned except for the peak relating to diffraction from the basal (002) plane of talc. For Talc\HWSS@Fe_3_O_4_, this peak slightly shifts and appears at 2θ = 9.97°. Calculation of d-spacing for the 002 plane shows that it decreases from 9.01 Å in pristine talc to 8.87 Å in Talc\HWSS@Fe_3_O_4_. Wang and co-workers assigned the peak of diffraction from the basal 002 plane as characteristic to talc interlayer spacing and showed that it disappears on complete exfoliation of talc^63^. This peak is seen with a decreased intensity in the XRD pattern of Talc\HWSS@Fe_3_O_4_ (Fig. 5), indicating that talc flakes were partially delaminated by HWSS@Fe_3_O_4_ NPs. Holland introduced a morphology index to quantify the crystallinity of talc using its peaks at 2θ values of diffractions from 004 and 020 planes^64^. Calculation of this index for the herein used talc and Talc\HWSS@Fe_3_O_4_ resulted in the values of 0.471 and 0.536, respectively. These indices reveal the dominant microcrystalline morphology of talc in both samples. Notably, the diffraction peaks of HWSS and Fe_3_O_4_ NPs are not detectable in the XRD-pattern of Talc\HWSS@Fe_3_O_4_ (see S2 in the SI), indicating that the amount and sizes of these two components are too small, in comparison with talc flakes, so give undetectable wide peaks of low intensity.

The EDX spectrum of Talc\HWSS@Fe_3_O_4_ displayed peaks at energies characteristic to all of its constituting elements. No elements other than C, O, Mg, Si, and Fe are detectable in this spectrum, featuring the reasonable purity of the composite (see S3 in the SI).

**Figure 6:**
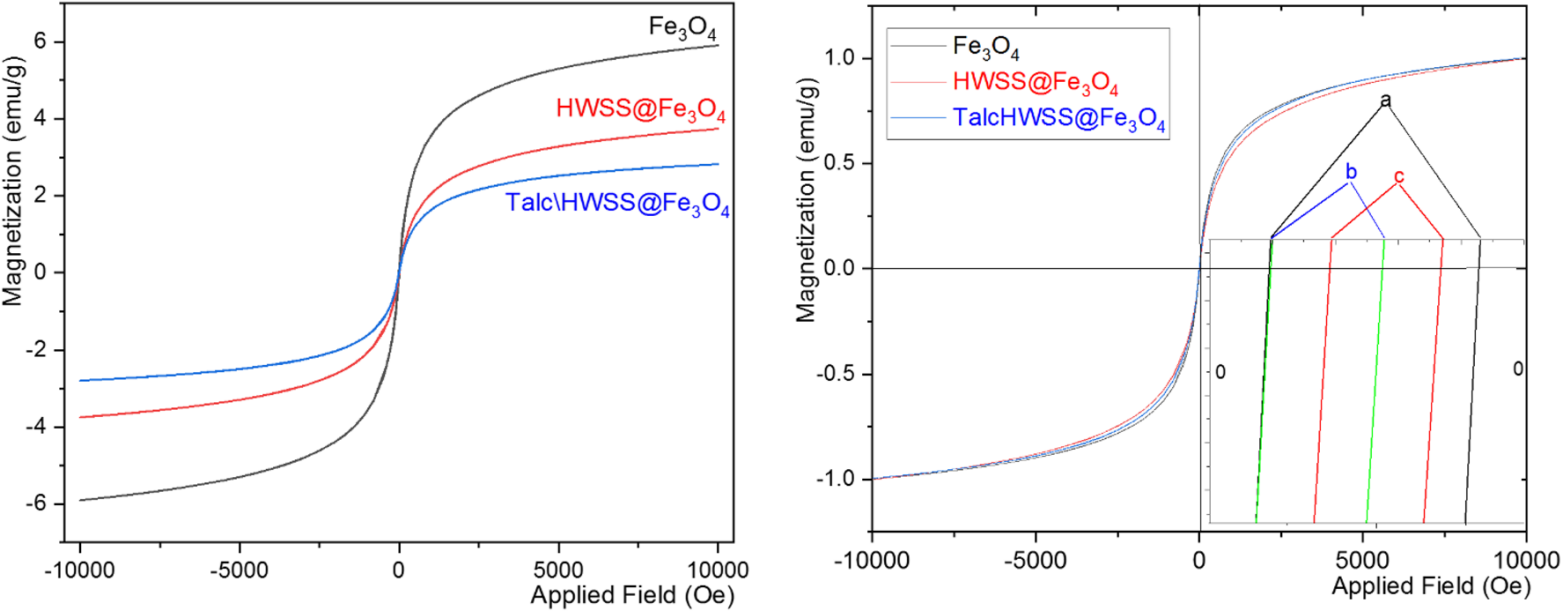
VSM curves (left) and the normalized VSM curves (right) of Fe_3_O_4_, HWSS@Fe_3_O_4_ and Talc\HWSS@Fe_3_O_4_. The inset shows the relative coercivities of the samples.

Magnetic behavior of the composite was studied by using a vibrating sample magnetometer. Figure 6 compares the magnetic saturation of Talc\HWSS@Fe_3_O_4_ with those of HWSS@Fe_3_O_4_ and Fe_3_O_4_. As this figure shows, mass saturation magnetization of Fe_3_O_4_ is decreased in the steps of its composition with HWSS and then with talc. For precise comparison, the VSM curves were normalized relative to their maximum magnetization values. The normalized curves of HWSS@Fe_3_O_4_ and Talc\HWSS@Fe_3_O_4_ have shown a nearly identical coercivity, which is about half the coercivity of the pristine Fe_3_O_4_. Based on this finding, talc has no effect on magnetic property of magnetite. Surface of a magnetite NP is prone to aerial oxidation and conversion to a Fe^+3^-enriched shell. Spin exchange of this shell with the inner zone of magnetite NP results in an increase of its coercivity. HWSS is conceived to reduce some Fe^+3^ atoms in the shell to Fe^+2^ atoms and this correction in stoichiometry of the shell would make the HWSS@Fe_3_O_4_ to have a smaller coercivity than Fe_3_O_4_ itself. Interestingly, the coercivity of HWSS@Fe_3_O_4_ NPs is preserved for a long time, indicating that Fe_3_O_4_ core in this composite has been efficiently protected from aerial oxidation by complexation with HWSS.

In order to qualify the adhesion of HWSS@Fe_3_O_4_ NPs to talc flakes, the Talc\HWSS@Fe_3_O_4_ nano-composite was alternatively prepared via namely path “b” through which the initially prepared HWSS@Fe_3_O_4_ NPs were refluxed with a suspension of talc (see S4 of SI). However, in the normal preparation pathway “a”, Fe_3_O_4_ NPs are prepared from a suspension of talc in Fe^2+^ and Fe^3+^ aqueous solution and then the suspension is refluxed with HWSS (Fig. 1). The VSM curves of the Talc\HWSS@Fe_3_O_4_ samples prepared via these two pathways were plotted in S5 (see SI).

According to these curves, the sample prepared from the normal pathway “a” showed a greater mass saturation magnetization than the one of path “b”. Both of these VSM curves displayed an identical coercivity of 12.4 Oe on normalization relative to their own maximum magnetization values. The supremacy of path “a” in delivering the composite with a greater mass magnetization value than path “b” lends credit to the argument that adhesion of HWSS and HWSS@Fe_3_O_4_ NPs to talc is facilitated by impregnation of talc surface with iron cations/hydroxides. That is, a higher density of magnetite NPs on talc is obtained from path “a”.

**Figure 7:**
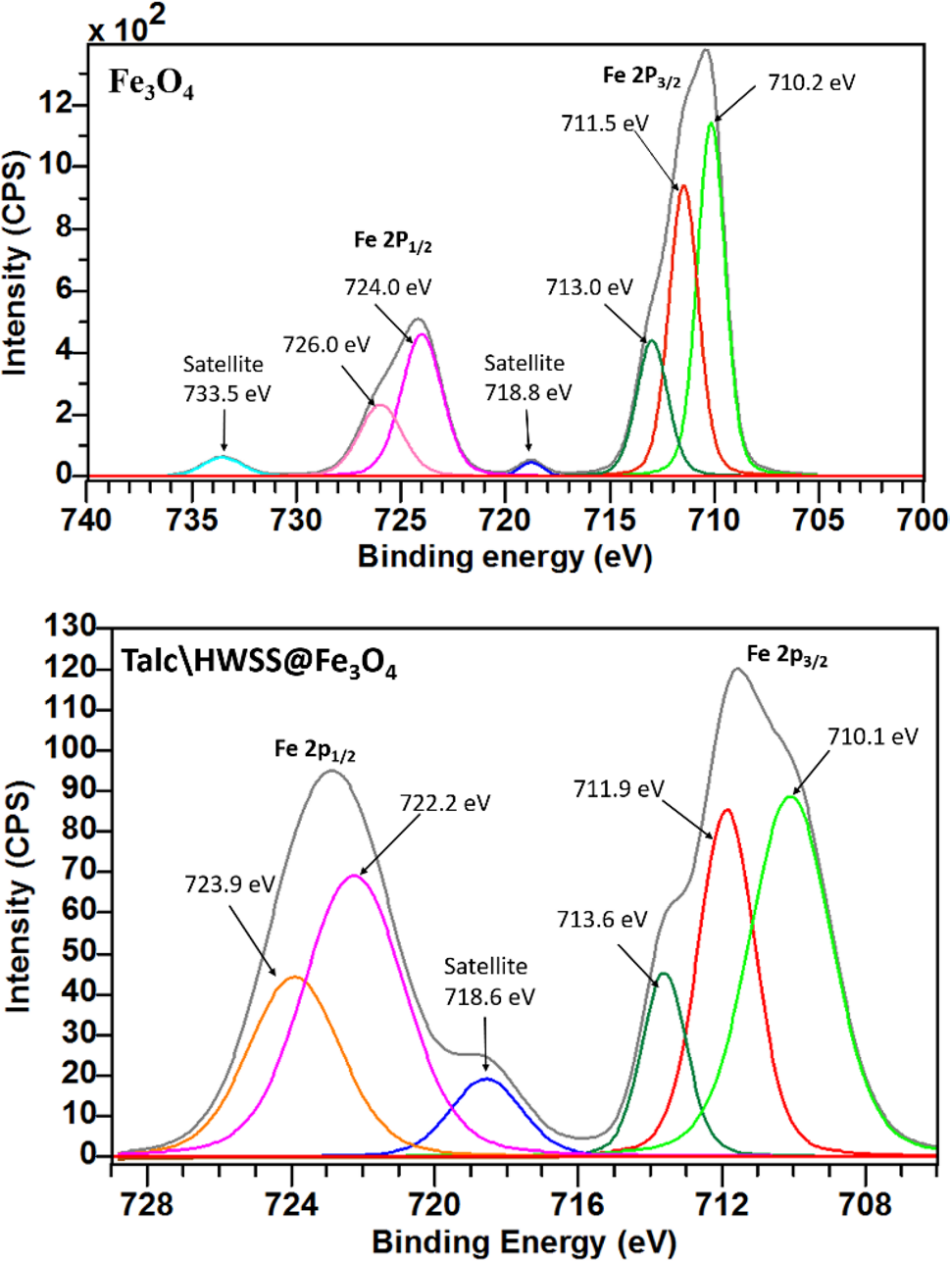
High resolution XPS spectra of Fe_3_O_4_ (top) and Talc\HWSS@Fe_3_O_4_ (down) in Fe 2p region.

The surface chemical composition of Talc\HWSS@Fe_3_O_4_ was examined by XPS. The survey spectrum showing the existence of Mg, C, Si, Fe, and O elements on the surface is shown in Fig. S7 (see the SI). The high resolution spectrum in the region of Mg_2p_ core level displays two components at 49.9 and 50.9 eV, which can be assigned to Mg^2+^ in Mg(OH)_2_ and O-Mg-O sites of talc, respectively (Fig. S8). The third component in this region at 51.6 eV corresponds to binding energy of the MgCO_3_ impurity formed by intake of atmospheric CO_2_ diffused into the alkalinized talc during production of the composite (Fig. S8)^65,66^. Figure S9 displays the high resolution core C_1s_ level, which can be fitted to allegedly three components, one strong feature at 285.1 eV corresponding to adventitious carbons and the C–C state of carbons in HWSS, a weak component at 283.9 eV attributing to C-H carbons, and a broad contribution from C–O, C=O, CO_2_ and CO_3_ carbons at 286.4 eV^67^. A nearly symmetric peak is detected in the O_1s_ region at 532.7 eV. Deconvolution of this peak gives the binding energies at 530.2, 532.5 and 534.2 eV, which account for the O_1s_ binding energy of metal oxides, C–OH, CO_2_H and CO_3_^−^ in the composite (Fig. S10)^68–71^. Detection of a symmetrical Lorentzian peak for binding energy of Si 2p at 103.6 eV means that the interactions with the silica layer of talc in the composite are rather hydrophobic (Fig. S11). A high resolution Fe 2p spectrum of Fe_3_O_4_ (shown in Fig. 7) consists of a doublet for Fe 2p_3/2_ at 711.5 eV and Fe 2p_1/2_ at 724.0 eV characteristic to Fe^3+^ atoms in octahedral sites. Another doublet of this spectrum at 713.0 eV for 2p_3/2_ and 726.0 eV for 2p_1/2_ is assigned to Fe^3+^ atoms in tetrahedral crystalline sites of magnetite. These doublets are accompanied with two shake up satellites at 718.8 and 733.5 eV. The 2p binding energy component at 710.2 eV or perhaps a part of it is ascribed to Fe^+2^ atoms and the other component from these atoms seem to be overlapped with the component at 724 eV^72,73^. Fe 2p region in high resolution XPS spectrum of Talc\HWSS@Fe_3_O_4_ displays similar features on fitting except that the components taking contributions from Fe^+2^ 2p binding energies gain an increased intensity. Due to these new features the two Fe 2p main peaks of Talc\HWSS@Fe_3_O_4_ become more asymmetric, as their intensities tail toward the lower binding energies. This finding suggests a significant interaction of HWSS with magnetite, whereby some surface Fe^+3^ to Fe^+2^ reduction is occurred.

Because of being a compact of known active components, the nano-composite seemed to act as an efficient catalyst. Hence, in the next phase of this investigation we planned to examine its catalytic activity in the synthesis of imidazo[1,2-*c*]quinazoline derivatives via the three-component Groebke-Blackburn-Bienaymé reaction. For this aim, the reaction of 4-aminoquinazoline **1a** with 4-chlorobenzaldehyde **2a** and cyclohexylisocyanide **3** was chosen as the model to be tested in various solvents, temperatures and in the presence of different amounts of the nano-composite. To check the significance of the results, the model reaction was also run in parallel experiments using some other catalysts.

The results of these optimization experiments were gathered in Table 1. As can be deduced from this table, the trial reaction gives the best yield under solvent-free conditions and proceeds smoothly in the presence of Talc/Fe_3_O_4_@HWSS to afford the desired product, 2-(4-chlorophenyl)-3-cyclohexylaminoimidazo[1,2-*c*]quinazoline **4a**, within 24 min (entry 12). Note that no product is formed within a reasonable time in the absence of any catalyst (entry 1). With other silica based and Lewis-acidic catalysts, the reaction yields were rather low within comparable reaction times (entries 2–5). However, montmorilonite and talc, i.e. two prevalent silica-derived minerals, are exceptions of Table 1, which have shown efficient catalytic activities in the model reaction (entries 6 and 7). Considering the negligible yield of the model reaction in the presence of MgO (entry 8), one may conclude that the reaction is not effectively catalyzed by bases. Solvents show negative effects on yield of the reaction even in the presence of Talc\HWSS@Fe_3_O_4_ (entries 10 and 11). Increasing the reaction temperature to above 120 °C leads to a decrease of yield, due to side reactions of the substrates. Monitoring the yield of the model product revealed that it reaches to a maximum by increasing the amount of the catalyst up to the optimum value of 40 mg per 1 mmol of each reactant. A comparison between the entries 5, 7 and 12 of Table 1 makes it evident that talc is the main catalytic component of Talc\HWSS@Fe_3_O_4_. Therefore, the slightly higher catalytic efficiency of Talc\HWSS@Fe_3_O_4_ relative to that of pristine talc can be ascribed to delamination and better dispersion of talc platelets in the composite.

**Table 1:** Optimization of the reaction conditions for the synthesis of **4a.**


Entry^a^	Catalyst	Solvent^b^	Temp. (°C)	Time (min)	Yield^c^ (%)
1	–	–	120	480	–
2	Zeolite-Y	–	100	30	39
3	MCM-41	–	100	30	55
4	TiO_2_	–	100	30	47
5	Fe_3_O_4_ NPs	–	100	30	35
6	Montmorilonite K10	–	100	30	71
7	Talc	–	100	30	69
8	MgO	–	100	30	trace
9	Talc\HWSS@Fe_3_O_4_	–	100	30	73
10	Talc\HWSS@Fe_3_O_4_	DMF	100	30	36
11	Talc\HWSS@Fe_3_O_4_	Et-OH	Reflux	30	23
12	Talc\HWSS@Fe_3_O_4_	–	**120**	**24**	**77**
13	Talc\HWSS@Fe_3_O_4_	–	130	24	74
14	Talc\HWSS@Fe_3_O_4_^d^	–	120	24	72
15	Talc\HWSS@Fe_3_O_4_^e^	–	120	24	77

^a^1 mmol of each substrate and 40 mg of a catalyst was used (otherwise stated). The optimal condition is in bold.

^b^5 mL of solvent (if any) was used.

^c^Isolated yields.

^d^30 mg of the catalyst was used.

^e^50 mg of the catalyst was used.

Impressed by these preliminary results, we set out to explore the scope of arylaldehydes tolerating the synthetic method set forth above for the model reaction. In this aiming, the optimal reaction conditions were applied to the synthesis of imidazo[1,2-*c*]quinazolines from the reaction of arylaldehydes with 4-aminoqunazoline and cyclohexylisocyanide. As shown in Table 2, all the utilized arylaldehydes expediently gave the corresponding products in fairly high yields and within short reaction times. It was found that aromatic aldehydes containing either electron-donating or electron-withdrawing groups react with nearly the same rate to deliver the desired imidazo[1,2-*c*]quinazolines in fairly high yields. All the synthesized products **4a–4g** are new compounds whose structures were elucidated from their IR, ^1^H NMR, ^13^C NMR and mass spectral data as well as elemental microanalysis.

**Table 2:** Synthesis of imidazo[1,2-*c*]quinazoline derivatives under the optimized conditions.


Product	R	Ar	Time (min)	Yield^a^ (%)		M.p. (°C)
**4a**	H	4-Cl·C_6_H_4_	24	77	143–145	
**4b**	H	4-F·C_6_H_4_	25	83	172–174	
**4c**	H	4-CH_3_O·C_6_H_4_	25	73	173–176	
**4d**	H	3-Cl·C_6_H_4_	24	74	141–143	
**4e**	CH_3_	4-C_3_H_7_·C_6_H_4_	30	68	143–146	
**4f**	H	4-C_3_H_7_·C_6_H_4_	30	78	183–186	
**4g**	H	2,4-Cl_2_·C_6_H_4_	30	72	154–156	

Reaction conditions: 1 mmol of each reactant, 40 mg of the catalyst and no solvent at 120 °C.

^a^Isolated yields.

A green aspect of the present method is the easily separation of the catalyst from reaction mixture by using a permanent magnet. In order to qualify this aspect of the method, the reusability of Talc\HWSS@Fe_3_O_4_ in the synthesis of compound **4a** was evaluated. After completion of the model reaction, CH_2_Cl_2_ was added to the hot reaction mixture and the solid catalyst was separated from the hot CH_2_Cl_2_ solution by using a permanent magnet, washed with ethanol and then dried before reuse in the next cycle of the model reaction. As Fig. S6 shows (see the SI), the nano-composite appreciably preserved its catalytic activity even after five times consecutive recycling in the model reaction.

**Figure 8:**
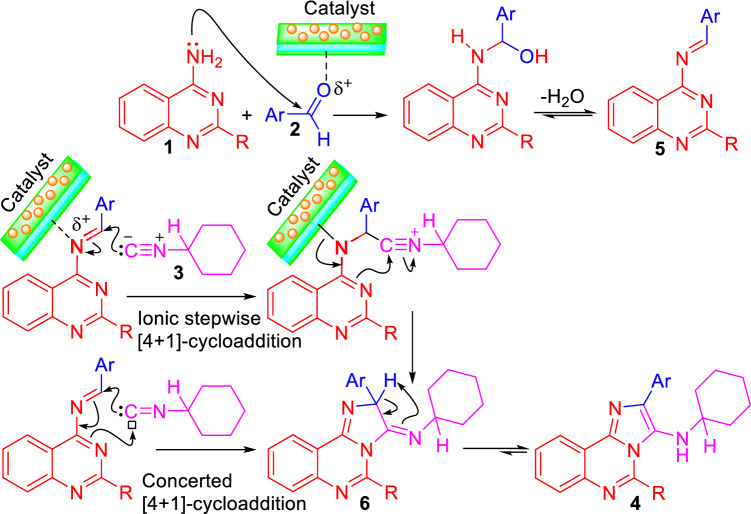
A mechanistic proposal for the synthesis of imidazo[1,2-*c*]quinazolines under catalysis of Talc\HWSS@Fe_3_O_4_.

Although, detailed experiments are required to verify the catalytic role of Talc\HWSS@Fe_3_O_4_ in the synthesis of **4**, a plausible pathway, in analogy with the previously presented mechanisms, is suggested in Fig. 8^74–78^. Very likely, the reaction goes through formation of the anil **5** from condensation of 4-aminoquinazoline **1** and the arylaldehyde **2**. Talc\HWSS@Fe_3_O_4_ seems to catalyze this condensation reaction by activating the aldehyde toward nucleophilic attack of 4-aminoquinazoline via coordination at its talc edges with the oxygen atom of the aldehydic carbonyl group. Next, the anil **5** undergoes a cycloaddition reaction with the isocyanide **3** to give the intermediate **6**. The cycloaddition reaction may proceed via an ionic stepwise path, initiating by nucleophilic addition of isocyanide **3** onto the electrophilic anil **5**, which has already been activated by coordination with the catalyst. Alternatively, the intermediate **6** may be formed from anil **5** and isocyanide **3** via a concerted [1 + 4] cycloaddition reaction. Because of its concerted nature, the latter cycloaddition mechanism is expected to be less affected by Talc\HWSS@Fe_3_O_4_. Tautomerization of the intermediate **6** leads to formation of the product **4**.

## Experimental

### Materials and methods

All the chemicals were purchased from Merck and Aldrich companies. Melting points were measured on an Electrothermal apparatus and are uncorrected. Transmission FT-IR spectra were taken in KBr pellets on a Shimadzu 8300 spectrometer. Attenuated total reflection (ATR) measurements were collected using a JASCO 4700 FT-IR spectrometer (Japan) equipped with a single-reflection Ge internal reflection element and a TGS detector. A layer of sample with ~ 0.2 mm thickness was loaded on the ATR surface by uniform pressure. Each spectrum was obtained from 16 scans in the spectral range of 400–4000 cm^–1^ and has a resolution of 2 cm^–1^. ^1^H- and ^13^C NMR spectra were recorded on a Bruker DRX-500 AVANCE instrument in CDCl_3_ as solvent at 500.1 and 125.7 MHz, respectively. Chemical shifts of ^1^H- and ^13^C NMR spectra were expressed in ppm downfield from TMS. Mass spectra were recorded on Shimadzu QP1100EX mass spectrometer operating at an ionization potential of 70 eV. Elemental analyses for C, H and N were performed using a Foss Heraus CHN-O-rapid analyzer. The transmission electron microscopy (TEM) images were recorded on a Philips EM208S, equipped with an energy dispersive X-ray spectrometer (EDX), at acceleration voltage of 100 kV. The X-ray powder diffraction (XRD) patterns were obtained using a XRD PW1730 X-ray diffractometer using Cu‐K_α_ radiation source of wavelength 1.540598 Å. Magnetic susceptibility measurements were performed by a VSMJDM‐13 Vibrating Sample Magnetometer at room temperature. The normalized VSM curves were obtained by plotting the relative M_s_/M_max_ values of samples versus the applied magnetic field (Oe); where M_s_ refers to the magnetic susceptibility of the sample at a given applied filed and M_max_ stands for its maximum susceptibility in the range of the applied magnetic fields. X-ray photoelectron spectra (XPS) were recorded with a Bes Tek (Germany) spectrometer using Al-Kα monochromatic radiation (1486.7 eV) under an operating pressure of 10^−10^ mbar. The binding energies were referenced by setting the adventitious carbon C 1s peak to 285 eV. The high-resolution spectra in the Fe 2p, O 1s, C 1s, Si 2p, and Mg 2p regions were fitted using CasaXPS with Gaussian/Lorentzian line shapes.

### Preparation of the nano-composite (Talc\HWSS@Fe_3_O_4_ NPs)

Wheat starch (0.5 g) was wetted with redistilled water (35 mL) in a 100 mL conical flask. The resultant slurry was heated at ~ 98 °C for 15 min with constant stirring. For separation of hot-water-insoluble starch (HWIS) from the supernatant solution of the HWSS fraction, the resulting suspension was centrifuged at 3000 rpm for 5 min.

For the synthesis of Talc\HWSS@Fe_3_O_4_ nano-composite, a mixture of FeCl_2_·4H_2_O (0.5 g) and FeCl_3_ (0.78 g) was dissolved in a solution of HCl (12 M, 0.2 mL). To this solution of Fe^3+^ and Fe^2+^ ions was added a suspension of talc (1.0 g) in 70 mL deionized water. The combined liquid was stirred for 24 h to obtain talc flakes impregnated with Fe^3+^ and Fe^2+^ ions. After this time the suspension was added dropwise and with continuous stirring to a freshly prepared (62.5 mL) NaOH solution (1.5 M). The resulting suspension of talc and Fe_3_O_4_ NPs was mixed with the already prepared HWSS solution (vide supra) by rapid mechanical stirring. The combined fluid was then refluxed at 60 °C for 6 h. At this end, the resulting NPs of Talc\HWSS@Fe_3_O_4_ were collected by placing the vessel of their suspension on an external magnet (0.7 Tesla) and decanting the supernatant solution. The NPs were washed twice with ethanol (7 mL) and distilled water (10 mL), and then dried in an oven at 100 °C for 3h.

### General procedure for the synthesis of imidazo[1,2-*c*]quinazoline derivatives

Talc\HWSS@Fe_3_O_4_ NPs (40 mg) was added to a mixture of 4-aminoquinazoline (0.145 g, 1 mmol), an aromatic aldehyde (1 mmol) and cyclohexyl isocyanide (0.112 g, 1 mmol) in a 50 mL flask. The flask was equipped with a simple condenser and heated at 120 °C under magnetic stirring. At end of the reaction, as monitored by TLC on silica gel 60 F_254_, using ethyl acetate:n-hexane in 1:2 ratio as eluent, CH_2_Cl_2_ (2 × 10 mL) was added to the reaction mixture and the catalyst was separated from the hot CH_2_Cl_2_ solution. For this purpose, the hot flask was place about 5 min on a permanent magnet and the supernatant CH_2_Cl_2_ solution was separated by decantation. The combined CH_2_Cl_2_ solution was concentrated under reduced pressure and the viscous residue was chromatographed over silica gel (using ethyl acetate:n-hexane in 1:2 ratio, as eluent) to obtain the product **4**. The product was further purified by recrystallization in ethyl acetate.

### Selected physical and spectral data of the products

Atoms in molecules of the products were numbered as illustrated in the figure of Table 1 (vide supra).

#### 2-(4-Chlorophenyl)-3-cyclohexylamino-imidazo[1,2-*c*]quinazoline (4a)

White solid, Mp. 143–145 °C. IR (KBr, υ_max_) 3297, 3075, 2932, 1604, 1559, 1544 and 1491 cm^−1^. ^1^H NMR (500 MHz, CDCl_3_): δ_H_ 8.88 (1H, s, 5-H), 8.57 (1H, dd, *J* 7.2 and 1.6 Hz, 10-H), 8.03 (2H, d, *J* 8.4 Hz, 2ʹ-H and 6ʹ-H), 7.6–7.7 (2H, m, 8-H and 9-H), 7.95 (1H, d, *J* 7.6 Hz, 7-H), 7.46 (2H, d, *J* 8.4 Hz, 3́ -H and 5ʹ-H), 3.17 (1H, s, N–H), 3.01(1H, m, 1ʺ-H), 1.9–1.2 (10H, m, cyclohexyl) ppm. ^13^C NMR (125.7 MHz, CDCl_3_): δ_C_ 141.0, 139.0, 135.3 (C-5), 134.5, 133.4, 132.3, 130.9, 129.9, 128.9, 128.5, 128.3, 125.7, 122.4, 119.3, 57.6 (C-1ʺ), 34.2 (C-2ʺ and C-6ʺ), 25.6 (C-3ʺ and C-5ʺ), 24.8 (C-4ʺ) ppm. Mass: m/z 378 (M^+^, ^37^Cl, 13), (376 (M^+^, ^35^Cl, 32), 295 (M^+^-cyclohexyl, ^37^Cl, 17), 293 (M^+^-cyclohexyl, ^35^Cl, 23), 183 (38), 167 (38), 129 (17), 83 (34), 57 (100). Anal. Calculated for (C_22_H_21_ClN_4_): C 70.11; H 5.62; N 14.87%. Found: C 70.24; H 5.71; N 14.60%.

#### 2-(4-Fluorophenyl)-3-cyclohexylamino-imidazo[1,2-*c*]quinazoline (4b)

White solid, Mp. 172–174 °C. IR (KBr, υ_max_) 3286, 3075, 2923, 2852, 1604, 1556, 1502, 1371, 1367, 1224, 836, 763 cm^−1^. ^1^H NMR (500 MHz, CDCl_3_): δ_H_ 8.89 (1H, s, 5-H), 8.58 (1H, dd, *J* 7.8, 1.5 Hz, 10-H), 8.06 (2H, dd, *J* 8.7, 5.5 Hz, 2ʹ-H and 6ʹ-H), 7.97 (1H, d, *J* 7.6 Hz, 7-H), 7.70 (1H, t, *J* 7.6 Hz), 7.66 (1H, t,* J* 7.6 Hz), 7.20 (2H, t, *J* 8.7 Hz, 3ʹ-H and 5ʹ-H), 3.19 (1H, d, *J* 4.9 Hz, NH), 3.0 (1H, m, 1ʺ-H), 1.9–1.2 (10H, m, cyclohexyl) ppm. ^13^C NMR (125.7 MHz, CDCl_3_): δ_C_ 162.7 (C-4ʹ, d, ^1^*J*_C-F_ 247 Hz), 130.4 (C-1ʹ, d, ^4^*J*_C-F_ 3 Hz), 129.3 (C-2ʹ and C-6ʹ d, ^3^*J*_C-F_ 8 Hz), 128.8 (C-3ʹ and C-5ʹ, d, ^2^*J*_C-F_ 11 Hz), 135.8 (C-5), 141.4, 139.3, 135.3, 130.2, 125.7, 122.8, 119.8, 116.1, 116.0, 58.0 (C-1ʺ), 34.6 (C-2ʺ and C-6ʺ), 25.2 (C-3ʺ and C-5ʺ), 26.0 (C-4ʺ) ppm. Mass: m/z 360 (M^+^, 8), 277 (M^+^-cyclohexyl, 18), 129 (100), 102 (54), 83 (46), 56 (100). Anal. Calculated for (C_22_H_21_FN_4_): C 73.31; H 5.87; N 15.54%. Found: C 73.42; H 5.93; N 15.39%.

#### 2-(4-Methoxyphenyl)-3-cyclohexylamino-imidazo[1,2-*c*]quinazoline (4c)

White solid, Mp. 172–176 °C. IR (KBr, υ_max_) 3344, 3075, 2923, 2850, 1602, 1564, 1506, 1386, 1211, 1244, 1022, 839 cm^−1^. ^1^H NMR (500 MHz, CDCl_3_): δ_H_ 8.92 (1H, s, 5-H), 8.60 (1H, dd, *J* 7.8, 1.5 Hz, 10-H), 8.01 (2H, d, *J* 8.7 Hz, 2́-H and 5́-H), 7.96 (1H, d, *J* 7.6 Hz, 7-H), 7.70 (1H, dt, *J* 7.8 and 1.5 Hz, 8-H), 7.66 (1H, t, *J* 7.8 Hz, 9-H), 7.05 (2H, d, *J* 8.7 Hz, 3ʹ-H and 5ʹ-H), 3.92 (3H, s, O-CH_3_), 3.21 (1H, s br, NH), 3.05 (1H, s br, 1ʺ-H), 1.2–1.9 (10H, m, cyclohexyl) ppm. ^13^C NMR (125.7 MHz, CDCl_3_): δ_C_ 159.6 (C-O), 141.4, 139.2, 136.0, 135.9 (C-5), 130.0, 128.8 (C-2ʹ and C-6ʹ), 128.7 (C-3ʹ and C-5ʹ), 126.8, 125.2, 122.8, 119.8, 114.6, 114.5, 58.0 (C-1ʺ), 55.7 (OCH_3_), 34.5 (C-2ʺ and C-6ʺ), 26.1 (C-4ʺ), 25.2 (C-3ʺ and C-5ʺ) ppm. Mass: m/z 372 (M^+^, 16), 289 (M^+^-cyclohexyl, 45), 129 (52), 102 (38), 83 (74), 67 (26), 56 (100). Anal. Calculated for (C_23_H_24_N_4_O): C 74.17; H 6.49; N 15.04%. Found: C 74.29; H 6.53; N 14.95%.

#### 2-(3-Chlorophenyl)-3-cyclohexylamino-imidazo[1,2-*c*]quinazoline (4d)

White solid, Mp. 141–143 °C. IR (KBr, υ_max_) 3325, 3075, 2925, 2849, 1595, 1569, 1473, 1367, 898, 769 cm^−1^. ^1^H NMR (500 MHz, CDCl_3_): δ_H_ 8.91 (1H, s, 5-H), 8.60 (1H, dd, *J* 7.6, 1.6 Hz, 10-H), 8.15 (1H, s, 2′-H), 7.98 (2H, d, *J* 7.8 Hz 7-H and 6́-H), 7.73 (1H, dt, *J* 7.6 and 1.6 Hz, 8-H), 7.68 (1H, t, *J* 7.6 Hz, 9-H), 7.44 (1H, t, *J* 7.9 Hz, 5́-H), 7.35 (1H, d, *J* 7.9 Hz, 4́-H), 3.22 (1H, s, N–H), 3.06 (1H, m, 1ʺ-H), 1.20–1.95 (10H, m, cyclohexyl) ppm. ^13^C NMR (125.7 MHz, CDCl_3_): δ_C_ 141.4, 139.4, 136.0, 135.8 (C-5), 135.1, 134.6, 130.3, 128.9, 128.8, 128.0, 127.6, 126.5, 125.4, 122.8, 119.8, 58.2 (C-1ʺ), 34.6 (C-2ʺ and C-6ʺ), 26.0 (C-3ʺ and C-5ʺ), 25.2 (C-4ʺ) ppm. Mass: m/z 376 (M^+^, ^35^Cl, 1), 295 (M^+^-cyclohexyl, ^37^Cl, 1), 293 (M^+^-cyclohexyl, ^35^Cl, 3), 129 (24), 102 (14), 83 (40), 67 (10), 56 (74). Anal. Calculated for (C_22_H_21_ClN_4_): C 70.11; H 5.62; N 14.87%. Found: C 70.18; H 5.71; N 14.75%.

#### 2-(4-Isopropylphenyl)-3-cyclohexylamino-5-methylimidazo[1,2-*c*]quinazoline (4e)

White solid, Mp. 143–146 °C. IR (KBr, υ_max_) 3315, 3075, 2964, 2927, 2850, 1616, 1560, 1386, 1330, 1263, 1076, 837, 761 cm^−1^. ^1^H NMR (500 MHz, CDCl_3_): δ_H_ 8.59 (1H, dd, *J* 7.9 and 1.4 Hz, 10-H), 7.88 (2H, d, *J* 8.2 Hz, 2ʹ-H and 6ʹ-H), 7.37 (2H, d, *J* 8.2 Hz, 3ʹ-H and 5ʹ-H), 7.84 (1H, d, *J* 8.0 Hz, 7-H), 7.64 (1H, dt, *J* 7.8 and 1.4 Hz, 8-H), 7.56 (1H, t, *J* 7.9 Hz, 9-H), 3.27 (1H, d, *J* 4.4 Hz, N–H), 3.21 (3H, s, 5-CH_3_), 3.03–3.00 (1H, m, Me_2_CH), 2.88 (1H, m, 1ʺ-H), 1.35 (6H, d, *J* 6.9 Hz, 2CH_3_), 1.8–1.13 (10H, m, cyclohexyl) ppm. ^13^C NMR (125.7 MHz, CDCl_3_): δ_C_ 148.8, 147.5, 141.0, 140.7, 132.1, 129.9, 128.1 (C-2ʹ and C-6ʹ), 127.8, 127.5, 127.4, 127.1 (C-3ʹ and C-5ʹ), 122.7, 119.4, 59.1 (C-1ʺ), 34.4 (C-2ʺ and C-6ʺ), 26.2 (C-4ʺ), 25.3 (C-3ʺ and C-5ʺ), 24.4 (2CH_3_), 23.2 (CH_3_) ppm. Mass: m/z 398 (M^+^, 4), 315 (M^+^-cyclohexyl, 10), 143 (100), 102 (66), 83 (100), 67 (50). Anal. Calculated for (C_26_H_30_N_4_): C 78.35; H 7.59; N 14.06%. Found: C 78.49; H 7.63; N 13.88%.

#### 2-(4-Isopropylphenyl)-3-cyclohexylaminoimidazo[1,2-*c*]quinazoline (4f)

White solid, Mp. 183–186 °C. IR (KBr, υ_max_) 3330, 3075, 2956, 2922, 2862, 1604, 1471, 1450, 1365, 892 cm^−1^. ^1^H NMR (500 MHz, CDCl_3_): δ_H_ 8.95 (1H, s, 5-H), 8.61 (1H, dd, *J* 7.7 Hz and 1.4 Hz, 10-H), 7.99 (2H, d, *J* 8.2 Hz, 2ʹ-H and 6ʹ-H), 7.97 (1H, d, *J* 8.0 Hz, 7-H), 7.70 (1H, dt, *J* 7.7 and 1.4 Hz, 8-H), 7.67 (1H, t, *J* 7.8 Hz, 9-H), 7.39 (2H, d, *J* 8.2 Hz, 3ʹ-H and 5ʹ-H), 3.26 (1H, d, *J* 5.0 Hz, NH), 3.12–3.07 (1H, m, 1ʺ-H), 3.02 (1H, sep, *J* 6.9 Hz, Me_2_CH), 1.35 (6H, d, *J* 6.9 Hz, 2CH_3_), 1.9–1.2 (10H, m, cyclohexyl) ppm. ^13^C NMR (125.7 MHz, DMSO-*d*_6_): δ_C_ 148.7, 147.0, 143.3, 141.0, 140.1, 127.6, 133.5, 129.6, 127.2, 127.0, 126.7, 126.4, 122.3, 119.1, 52.9 (C-1ʺ), 33.2, 32.1 (C-2ʺ and C-6ʺ), 24.4 (C-4ʺ), 23.8 (C-3ʺ and C-5ʺ), 23.5 (CH_3_) ppm. Mass: m/z 384 (M^+^, 1), 301 (M^+^-cyclohexyl, 12), 129 (100), 102 (82), 83 (100). Anal. Calculated for (C_25_H_28_N_4_): C 78.09; H 7.34; N 14.57%. Found: C 78.12; H 7.41; N 14.47%.

#### 2-(2,4-Dichlorophenyl)-3-cyclohexylaminoimidazo[1,2-*c*]quinazoline (4g)

White solid, Mp. 154–156 °C. IR (KBr, υ_max_) 3278, 3068, 2933, 2874, 1652, 1597, 1474, 1370 cm^−1^. ^1^H NMR (500 MHz, CDCl_3_): δ_H_ 8.95 (1H, s, 5-H), 8.54 (1H, dd, *J* 8.0 Hz and 1.6 Hz, 10-H), 7.74–7.64 (3H, m, 6́-H, 9-H and 8-H), 7.42 (1H, dd, *J* 8.4 and 2.0 Hz, 5́-H), 7.55 (1H, d, *J* 2.0 Hz, 3́-H), 7.98 (1H, dd, *J* 7.6 and 1.2 Hz, 7-H), 3.29 (1H, d, *J* 6.8 Hz, N–H), 2.77 (1H, m, 1ʺ-H), 1.96–1.2 (10H, m, cyclohexyl) ppm. ^13^C NMR (125.7 MHz, DMSO): δ_C_ 139.9, 136.2, 134.3, 133.8, 133.2, 132.7, 129.4, 128.8, 128.6, 128.5, 128.1, 127.6, 127.1, 121.4, 119.0, 54.9 (C-1ʺ), 32.9 (C-2ʺ and C-6ʺ), 25.2 (C-3ʺ and C-5ʺ), 24.2 (C-4ʺ) ppm. Mass: m/z 414 (M^+^, ^37^Cl, ^37^Cl, 4), 412 (M^+^, ^35^Cl, ^37^Cl, 10), 410 (M^+^, ^35^Cl, ^35^Cl, 16), 327 (M^+^-cyclohexyl, ^35^Cl, ^35^Cl, 12), 329 (M^+^-cyclohexyl, ^37^Cl, ^35^Cl, 9), 331 (M^+^-cyclohexyl, ^37^Cl, ^37^Cl, 8), 129 (30), 83 (39), 57 (100). Anal. Calculated for (C_22_H_20_Cl_2_N_4_): C 64.24; H 4.90; N 13.62%. Found: C 64.18; H 4.86; N 13.71%.

## Conclusion

Hot-water-soluble starch (HWSS) mediated an increase in loading of magnetite nanoparticles onto microcrystalline talc flakes and played as an adhesive in cementing the resulting conglomerate. Transmission FT-IR studies revealed a significant interaction between HWSS and the magnetite nanoparticles, which certainly results in encapsulation of magnetite nanoparticles by thin films of HWSS. Based on these FT-IR studies, the adsorption of HWSS onto talc sheets is due to hydrophobic interactions between these two components and can be enhanced by pre-impregnation of talc flakes with iron ions. These facts are the basis of the protocol developed here for increasing the loading of magnetite nanoparticles onto talc sheets. ATR FT-IR spectra of the as-prepared nano-composite displayed partial delamination of talc sheets by HWSS@Fe_3_O_4_ nanoparticles. XPS survey spectrum displayed the characteristic peaks of all the constituting elements of the composite. Fitting the high resolution XPS spectrum of the composite in the Fe_2p_ region and its deconvolution revealed the components contributed by the Fe^+2^ 2p binding energy and get intensified by formation of the composite. VSM measurements revealed an identical coercivity for both HWSS@Fe_3_O_4_ nanoparticles and Talc\HWSS@Fe_3_O_4_ nano-composite, which is about half the coercivity value of the pristine Fe_3_O_4_ sample they were prepared from. This observation means that a number of Fe^+3^ atoms generated by aerial oxidation in the outermost volume of Fe_3_O_4_ nanoparticles are reduced by HWSS to Fe^+2^ atoms. By this stoichiometric correction, the spin exchange between the inner zone of magnetite nanoparticle and its outer layer is diminished, resulting in a decrease of the coercivity. The TEM images showed that a majority of talc edges remain uncoated during the composition. This fact was interpreted as talc impregnation with iron ions and in turn coating with HWSS@Fe_3_O_4_ nanoparticles occurs preferably at its basal silica surface and slightly remote from the edges. As a result, the edges remain uncoated during the composition and remain freely accessible for the catalytic reaction. In practice, the nano-composite appeared as an efficient heterogeneous catalyst in the synthesis of imidazo[1,2-*c*]quinazolines via a domino three-component reaction of 4-aminoquinazoline, arylaldehydes and cyclohexyl isocyanide. The improved catalytic activity of Talc\HWSS@Fe_3_O_4_ in comparison to pristine talc can be attributed to delamination of talc by HWSS@Fe_3_O_4_ NPs and hence better dispersion of talc sheets in the nano-composite. It has a superparamagnetic behavior and proved to be easily reusable, after simple magnetic separation, without significant loss of its activity.

### Supplementary Information

Supplementary Figures.

## Data Availability

All the data generated or obtained from analyses of this study are included in the article and its supplementary information file.
