# Supplementary material for: Starch mediates and cements densely magnetite-coating of talc, giving an efficient nano-catalyst for three-component synthesis of imidazo[1,2-c]quinazolines
Source: Sci Rep. 2024 Jan 5;14:666. doi: 10.1038/s41598-023-51123-y (PMC10770035; doi:10.1038/s41598-023-51123-y)

**Starch mediates and cements densely magnetite-coating of talc, giving an efficient nano-catalyst for three-component synthesis of imidazo[1,2-*c*]quinazolines**

Hedyeh Hosseinzadeh, Kurosh Rad-Moghadam*, Morteza Mehrdad, and Somayyeh Rouhi

*Chemistry Department, University of Guilan, Rasht 41335-1914, Iran. E-mail* [*radmm@guilan.ac.ir*](mailto:radmm@guilan.ac.ir)

**Table of contents**

| **Figure S1.** Comparative ATR FT-IR spectra of Talc, Talc\HWSS, and Talc\HWSS@Fe_3_O_4_ |
| --- |
| **Figure S2.** XRD patterns of starch and magnetite |
| **Figure S3.** Energy dispersive X-ray (EDX) spectrum of Talc/HWSS@Fe_3_O_4_ NPs |
| **Figure S4.** Path “b” for production of Talc\HWSS@Fe_3_O_4_ NPs |
| **Figure S5.** VSM curves of the Talc\HWSS@Fe_3_O_4_ nano-composite prepared *via* paths a and b |
| **Figure S6.** Yields of the model product **4a** versus recycling times. The reaction time was set constant at 24 min and the yields are the mean values of triplicate experiments. |
| **Figure S7.** The XPS survey spectrum of Talc\HWSS@Fe_3_O_4_ NPs |
| **Figure S8.** The high resolution Mg_2p_ X-ray photoelectron spectrum |
| **Figure S9.** The high resolution C_1s_ X-ray photoelectron spectrum |
| **Figure S10.** The high resolution O_1s_ X-ray photoelectron spectrum |
| **Figure S11.** The high resolution Si_2p_ X-ray photoelectron spectrum |
| **Figure S12.** ^1^H NMR spectrum of 2-(4-chlorophenyl)-3-cyclohexylamino-imidazo[1,2-*c*]quinazoline **(4a)** |
| **Figure S13.** ^13^C NMR spectrum of 2-(4-chlorophenyl)-3-cyclohexylamino-imidazo[1,2-*c*]quinazoline **(4a)** |
| **Figure S14.** Mass spectrum of 2-(4-chlorophenyl)-3-cyclohexylamino-imidazo[1,2-*c*]quinazoline **(4a)** |
| **Figure S15.** ^1^H NMR spectrum of 2-(4-fluorophenyl)-3-cyclohexylamino-imidazo[1,2-*c*]quinazoline **(4b)** |
| **Figure S16.** ^13^C NMR spectrum of 2-(4-fluorophenyl)-3-cyclohexylamino-imidazo[1,2-*c*]quinazoline **(4b)** |
| **Figure S17.** Mass spectrum of 2-(4-fluorophenyl)-3-cyclohexylamino-imidazo[1,2-*c*]quinazoline **(4b)** |
| **Figure S18.** ^1^H NMR spectrum of 2-(4-methoxyphenyl)-3-cyclohexylamino-imidazo[1,2-*c*]quinazoline **(4c)** |
| **Figure S19.** ^13^C NMR spectrum of 2-(4-methoxyphenyl)-3-cyclohexylamino-imidazo[1,2-*c*]quinazoline **(4c)** |
| **Figure S20.** Mass spectrum of 2-(4-methoxyphenyl)-3-cyclohexylamino-imidazo[1,2-*c*]quinazoline **(4c)** |
| **Figure S21.** ^1^H NMR spectrum of 2-(3-chlorophenyl)-3-cyclohexylamino-imidazo[1,2-*c*]quinazoline **(4d)** |
| **Figure S22.** ^13^C NMR spectrum of 2-(3-chlorophenyl)-3-cyclohexylamino-imidazo[1,2-*c*]quinazoline **(4d)** |
| **Figure S23.** Mass spectrum of 2-(3-chlorophenyl)-3-cyclohexylamino-imidazo[1,2-*c*]quinazoline **(4d)** |
| **Figure S24.** ^1^H NMR spectrum of 2-(4-isopropylphenyl)-3-cyclohexylamino-5-methyl-imidazo[1,2-*c*]quinazoline **(4e)** |
| **Figure S25.** ^13^C NMR spectrum of 2-(4-isopropylphenyl)-3-cyclohexylamino-5-methyl-imidazo[1,2-*c*]quinazoline **(4e)** |
| **Figure S26.** Mass spectrum of 2-(4-isopropylphenyl)-3-cyclohexylamino-5-methyl-imidazo[1,2-*c*]quinazoline **(4e)** |
| **Figure S27.** ^1^H NMR spectrum of 2-(4-isopropylphenyl)-3-cyclohexylamino-imidazo[1,2-*c*]quinazoline **(4f)** |
| **Figure S28.** ^13^C NMR spectrum of 2-(4-isopropylphenyl)-3-cyclohexylamino-imidazo[1,2-*c*]quinazoline **(4f)** |
| **Figure S29.** Mass spectrum of 2-(4-isopropylphenyl)-3-cyclohexylamino-imidazo[1,2-*c*]quinazoline **(4f)**  **Figure S30.** ^1^H NMR spectrum of 2-(2,4-dichlorophenyl)-3-cyclohexylamino-imidazo[1,2-*c*]quinazoline **(4g)** |
| **Figure S31.** ^13^C NMR spectrum of 2-(2,4-dichlorophenyl)-3-cyclohexylamino-imidazo[1,2-*c*]quinazoline **(4g)** |
| **Figure S32.** Mass spectrum of 2-(2,4-dichlorophenyl)-3-cyclohexylamino-imidazo[1,2-*c*]quinazoline **(4g)** |

**Figure S1.** Comparative ATR FT-IR spectra of Talc, Talc\HWSS, and Talc\HWSS@Fe_3_O_4_


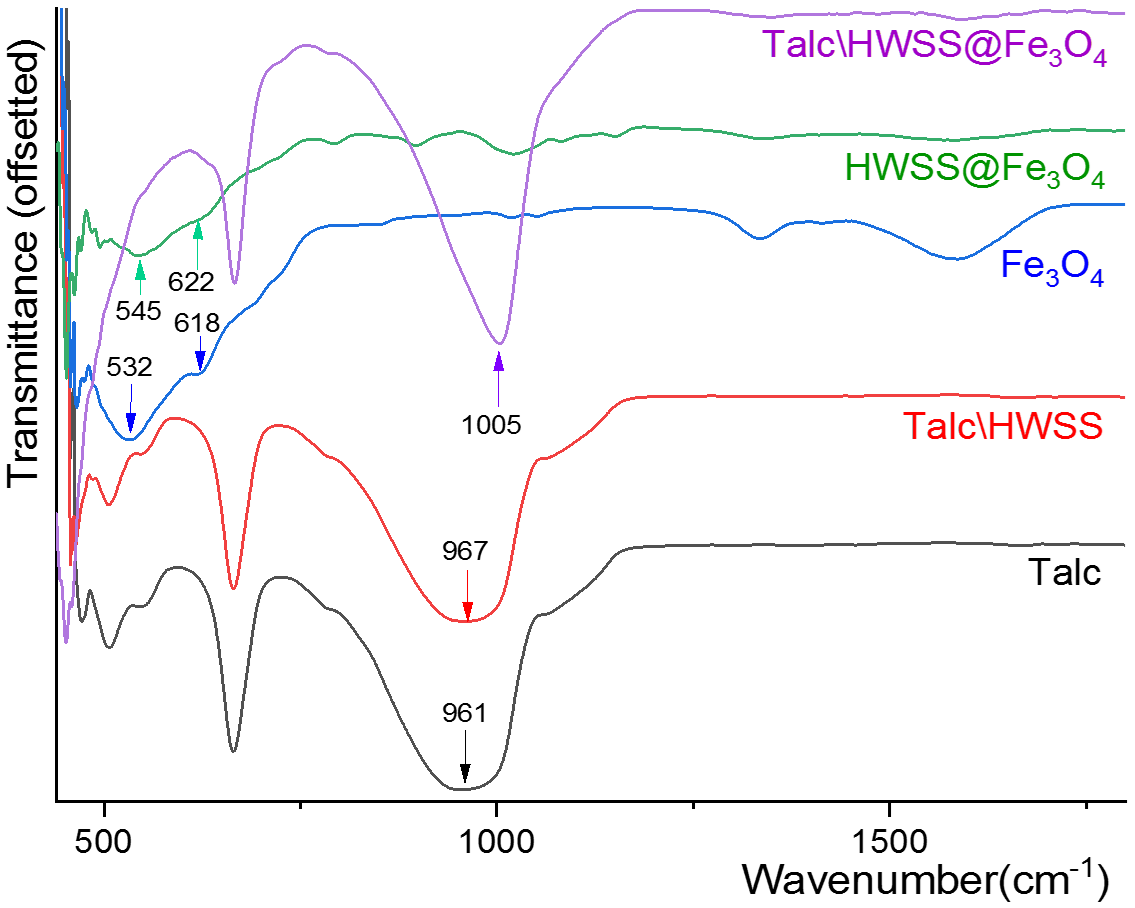


**Figure S2.** XRD patterns of starch and magnetite


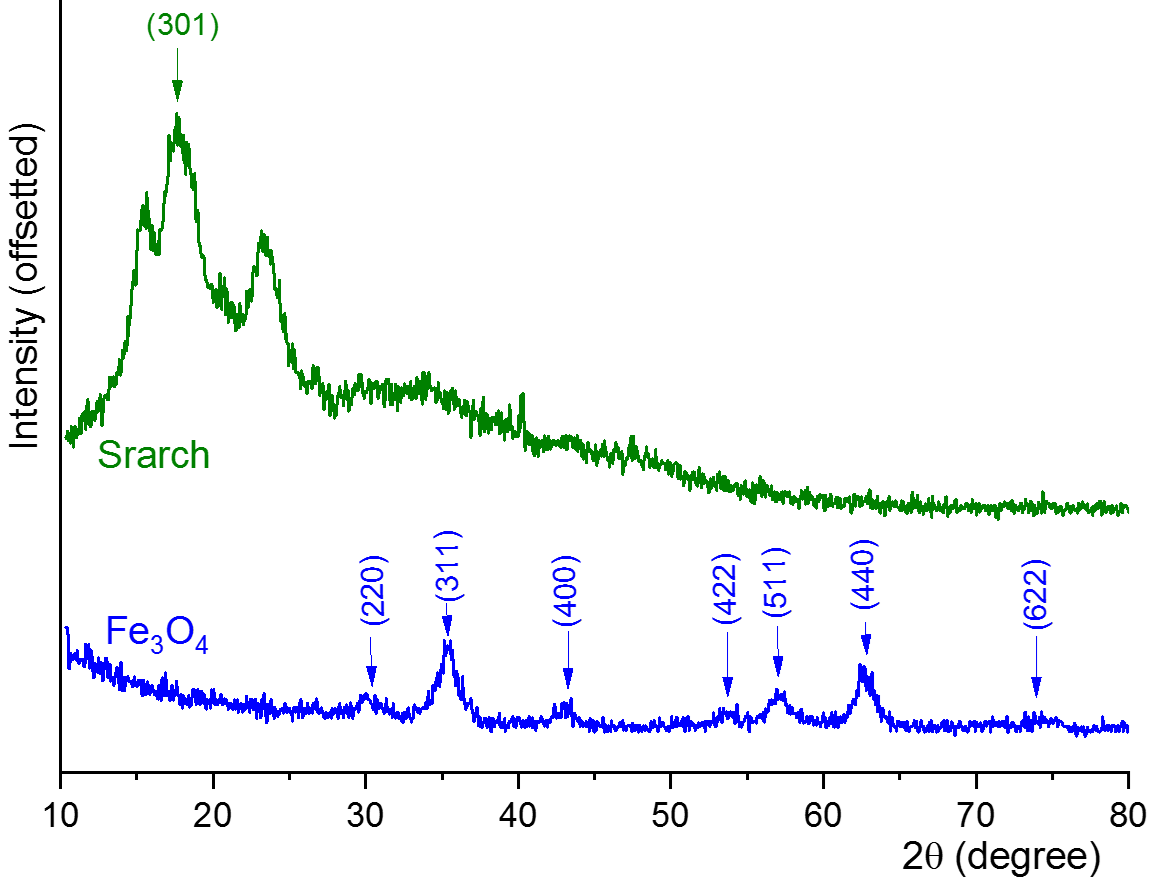


**Figure S3.** Energy dispersive X-ray (EDX) spectrum of Talc/HWSS@Fe_3_O_4_ NPs
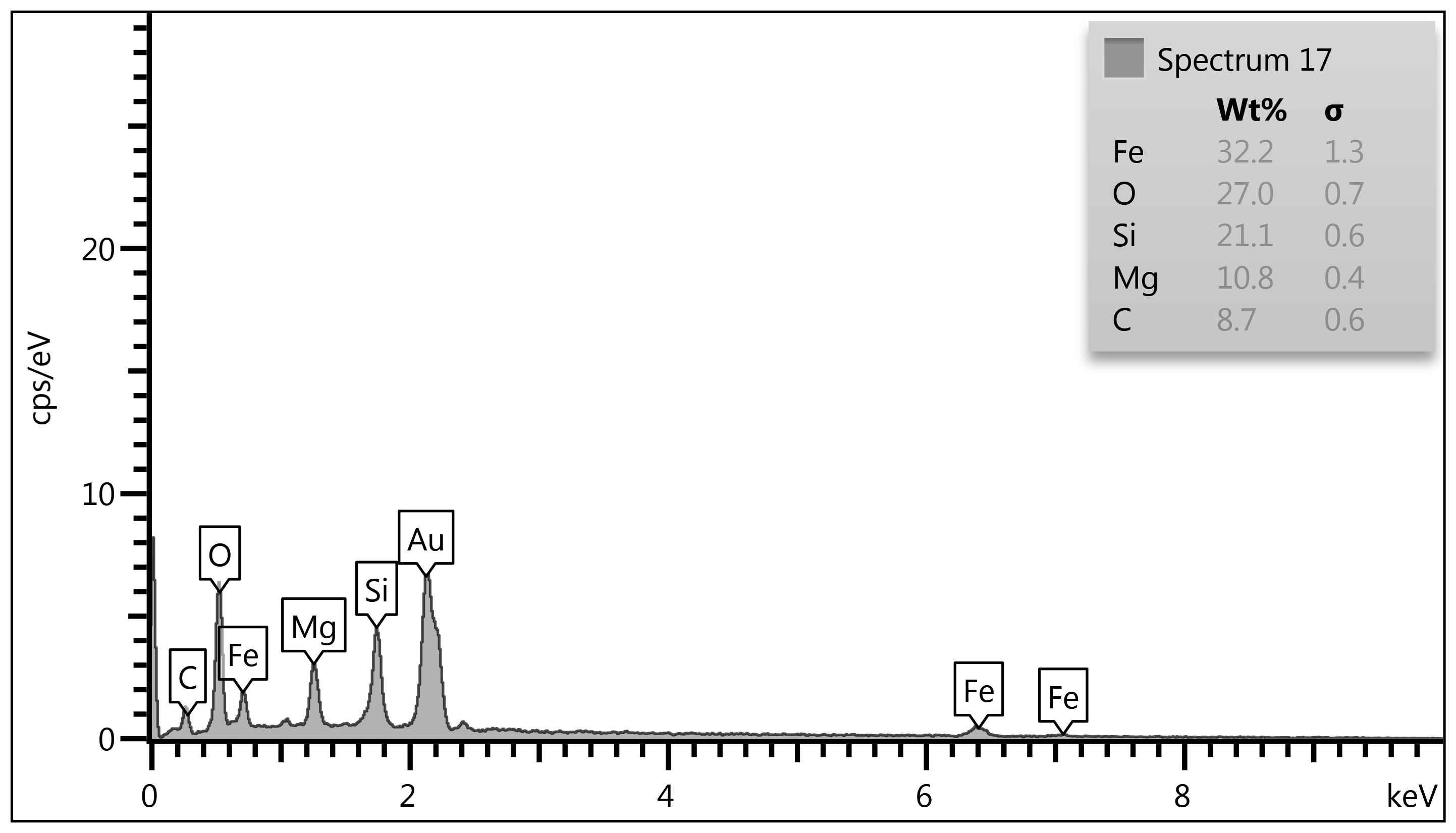


**Figure S4.** Path “b” for production of Talc\HWSS@Fe_3_O_4_ NPs

**Figure S5.** VSM curves of the Talc\HWSS@Fe_3_O_4_ nano-composite prepared *via* paths a and b


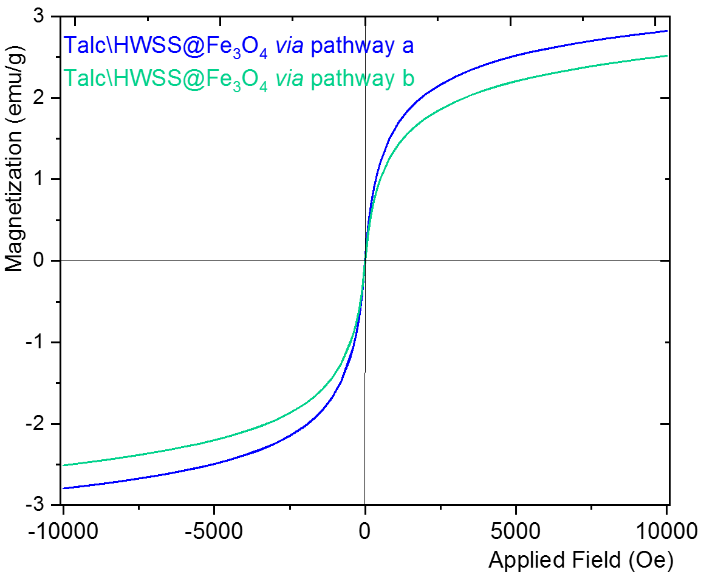


**Figure S6.** Yields of the model product **4a** versus recycling times. The reaction time was set constant at 24 min and the yields are the mean values of triplicate experiments.


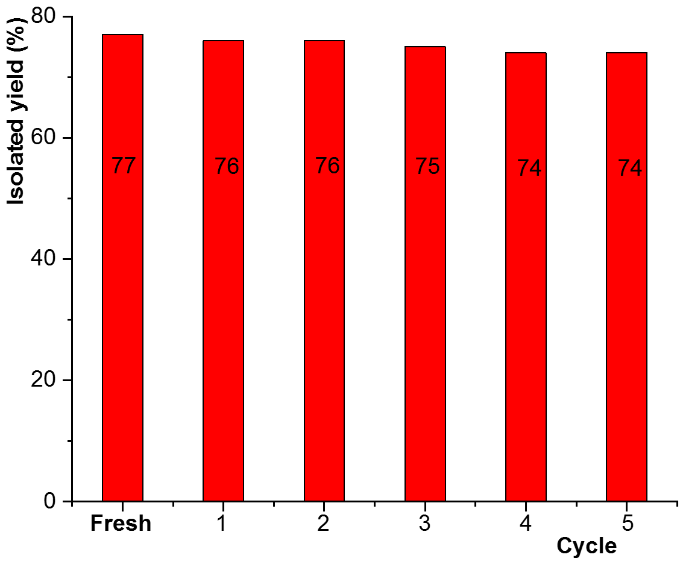


**Figure S7.** The XPS survey spectrum of Talc\HWSS@Fe_3_O_4_ NPs

**
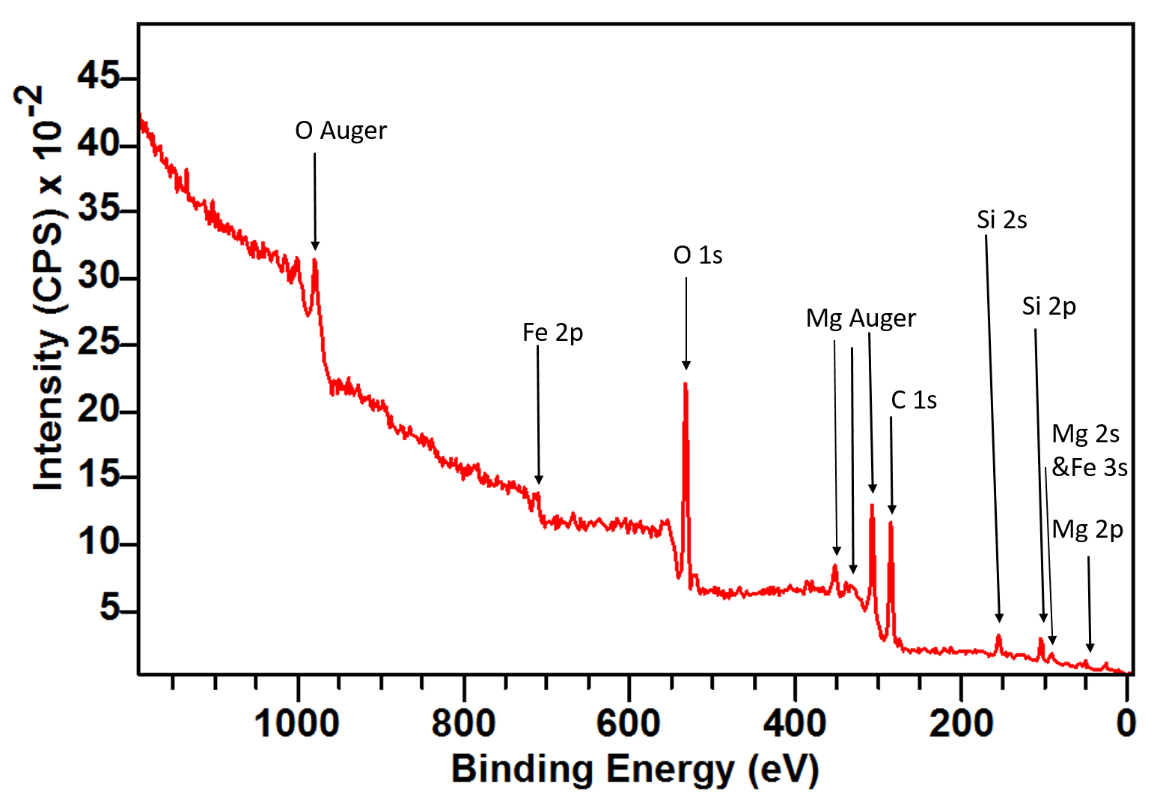
**

**Figure S8.** The high resolution Mg_2p_ X-ray photoelectron spectrum

**
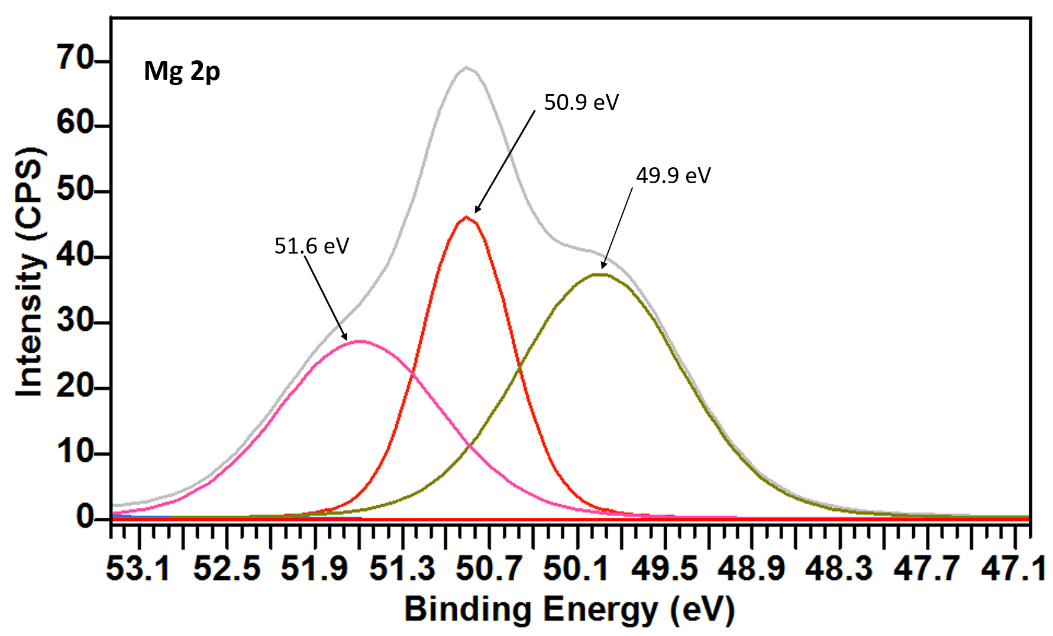
**

**Figure S9.** The high resolution C_1s_ X-ray photoelectron spectrum

**
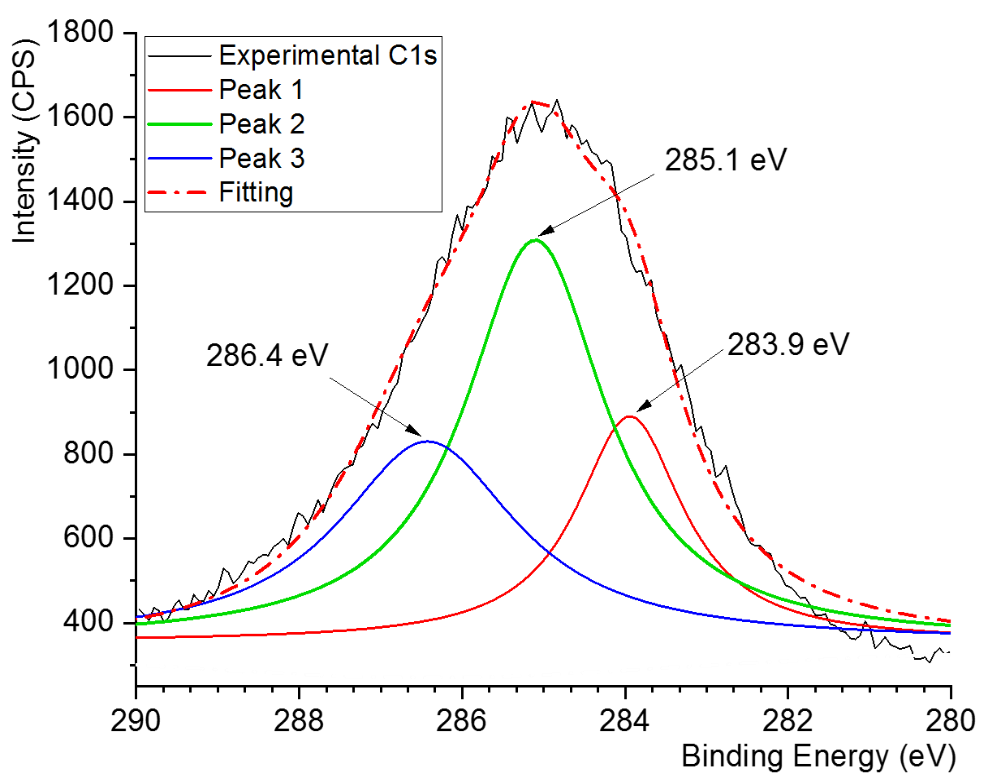
**

**Figure S10.** The high resolution O_1s_ X-ray photoelectron spectrum

**
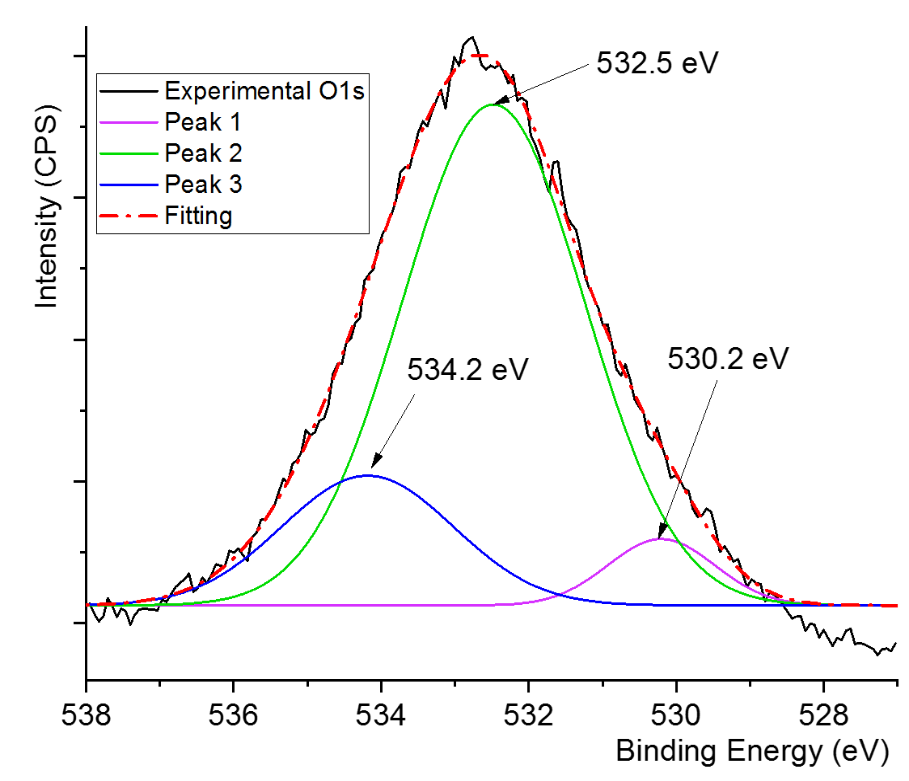
**

**Figure S11.** The high resolution Si_2p_ X-ray photoelectron spectrum

**
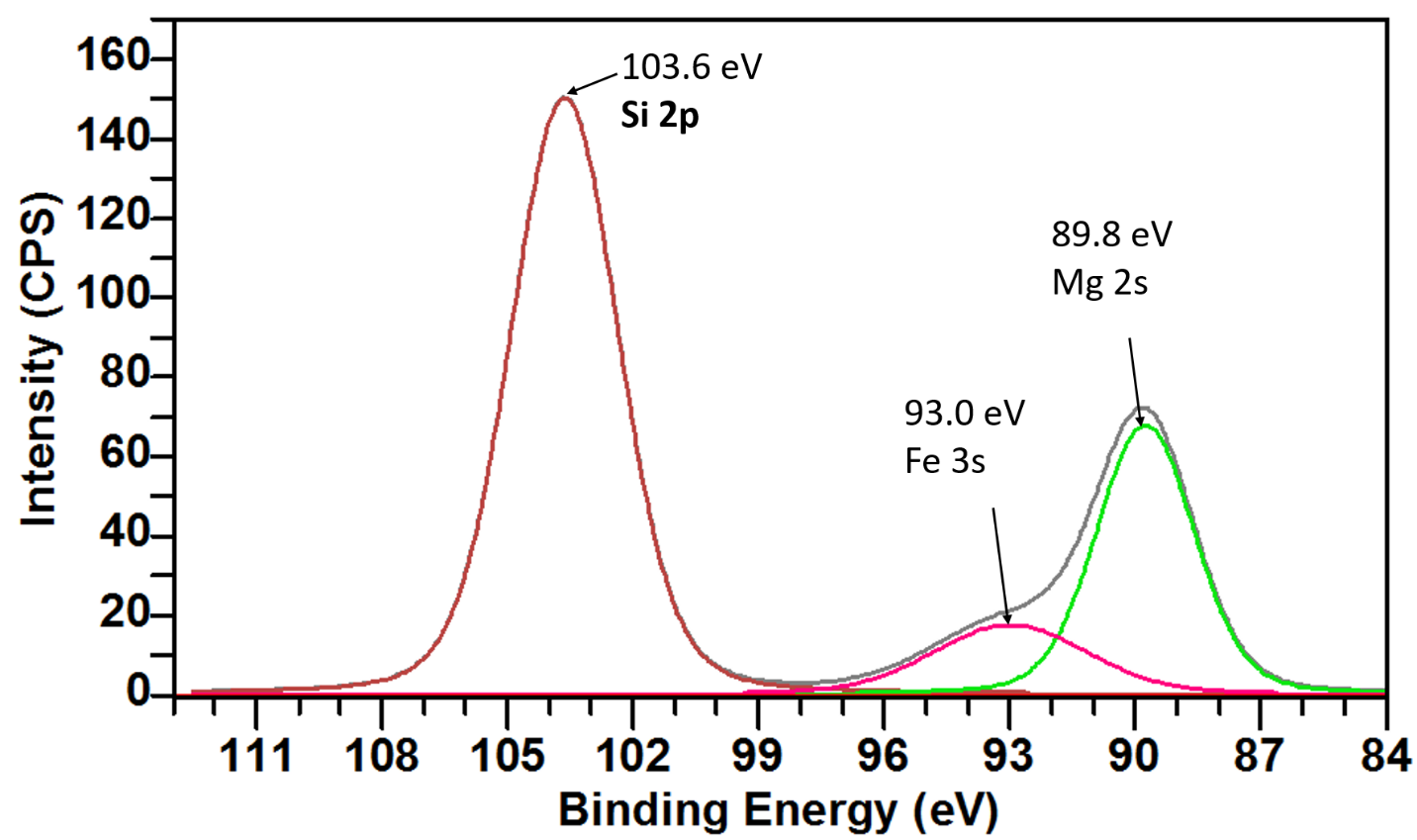
**

**Figure S12.** ^1^H NMR spectrum of 2-(4-chlorophenyl)-3-cyclohexylamino-imidazo[1,2-*c*]quinazoline **(4a)**


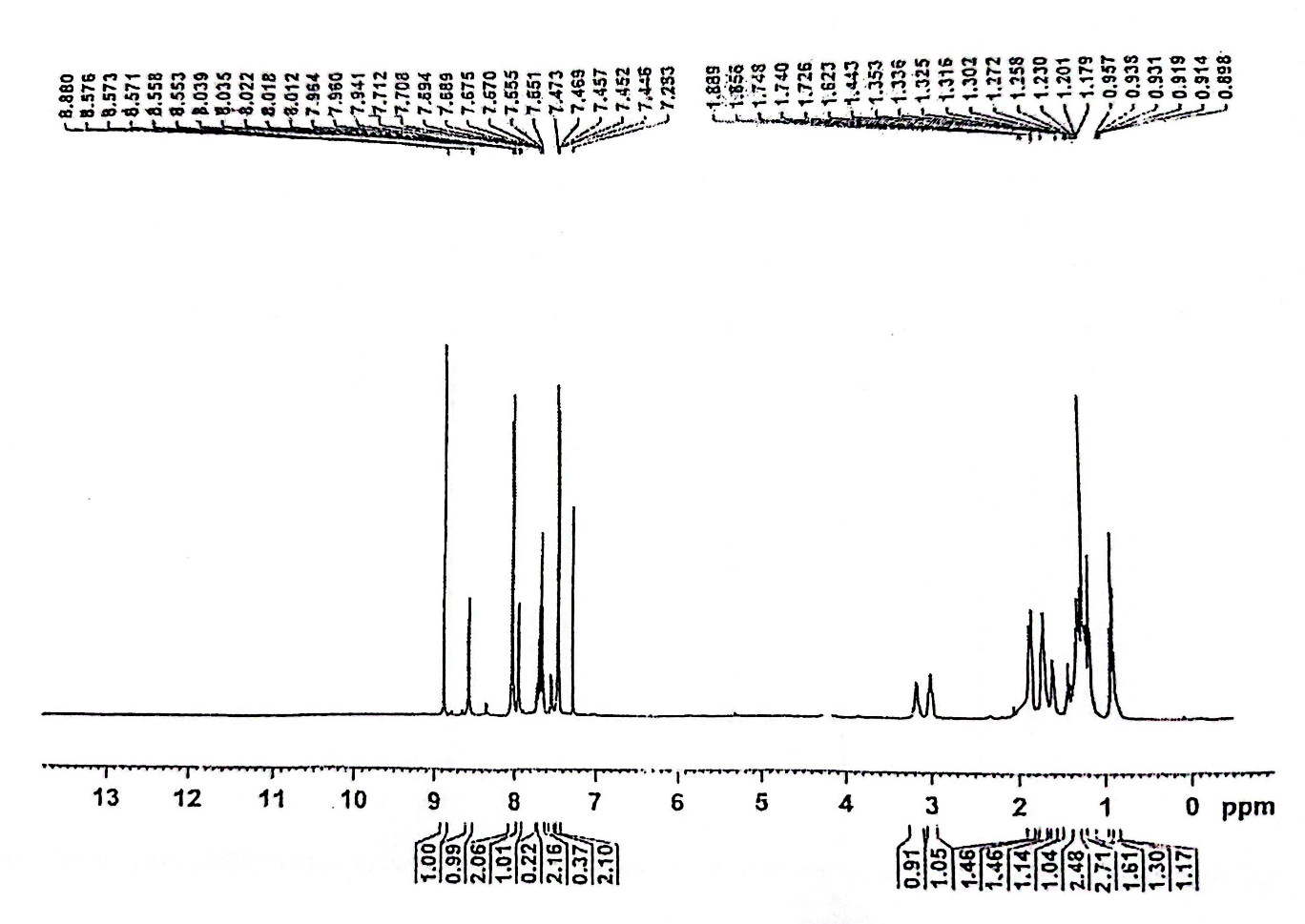


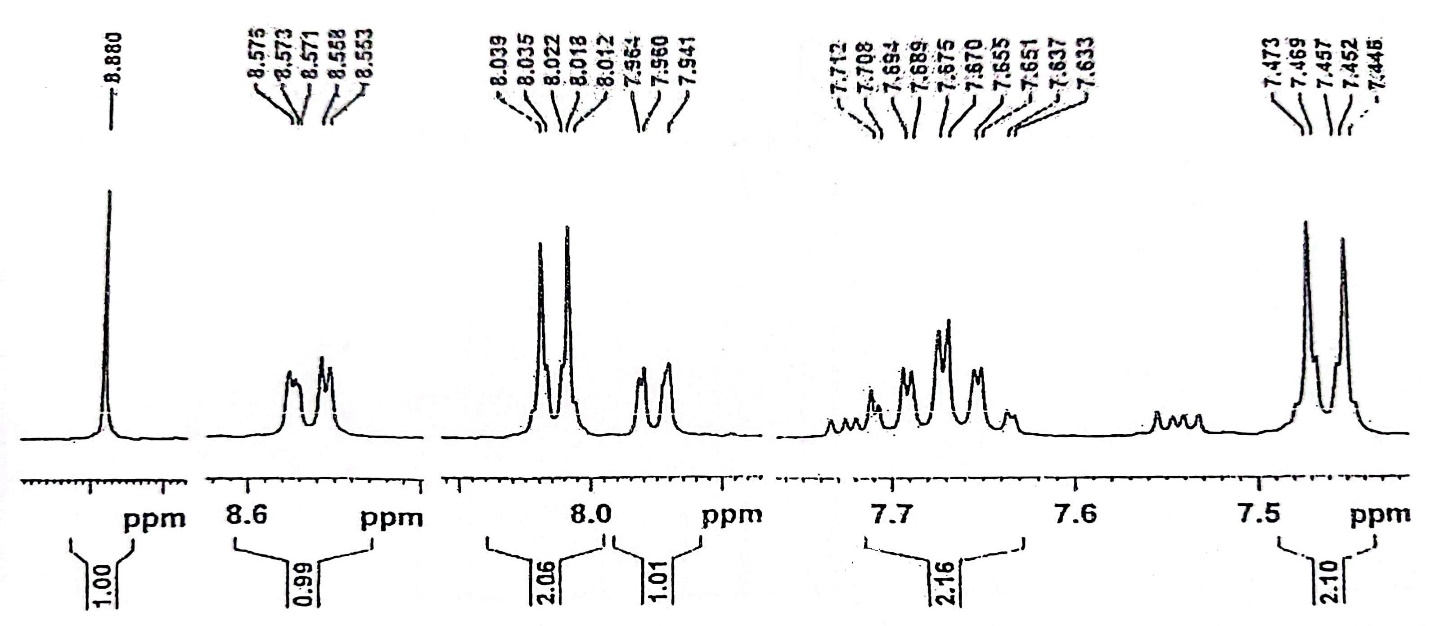


**Figure S13.** ^13^C NMR spectrum of 2-(4-chlorophenyl)-3-cyclohexylamino-imidazo[1,2-*c*]quinazoline **(4a)**


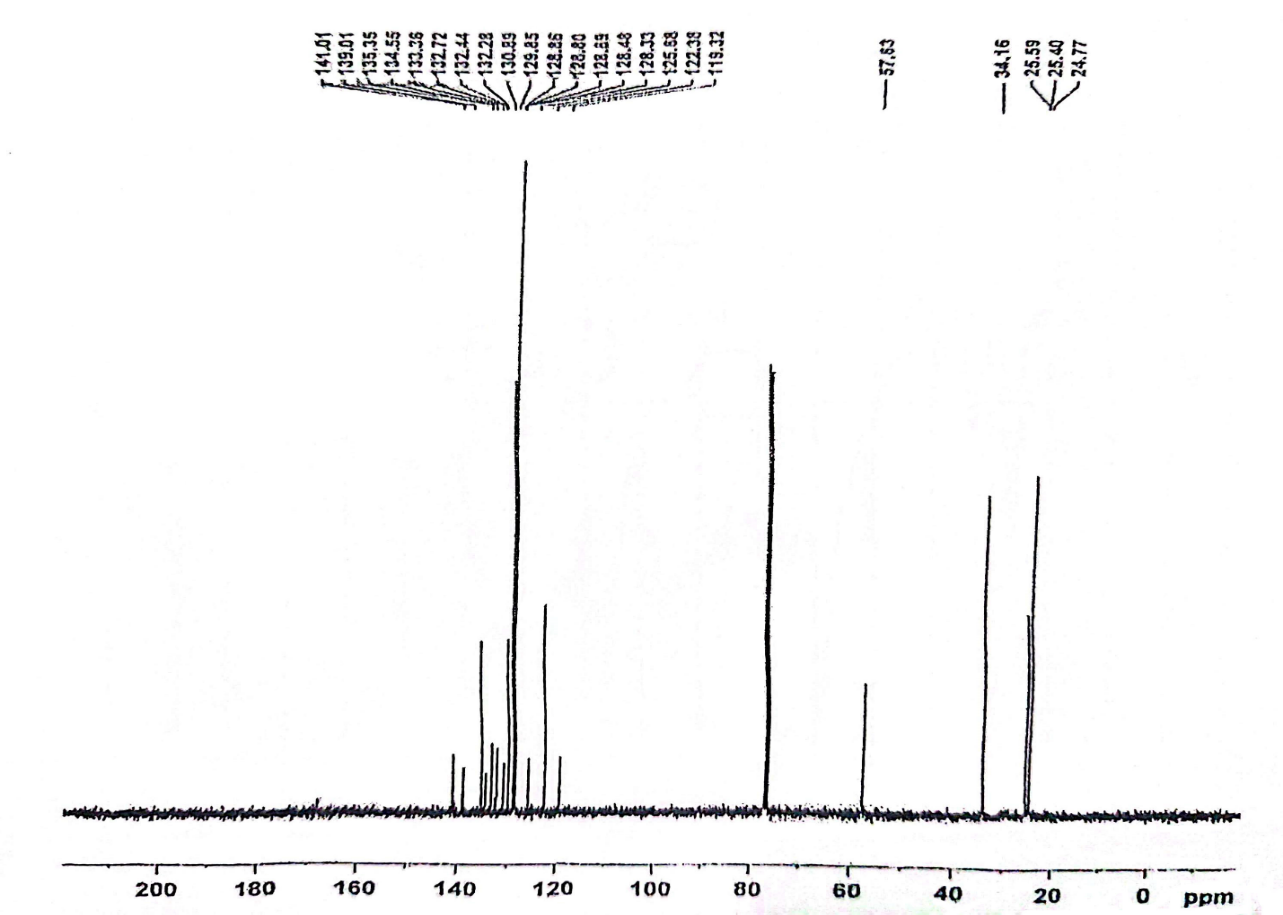


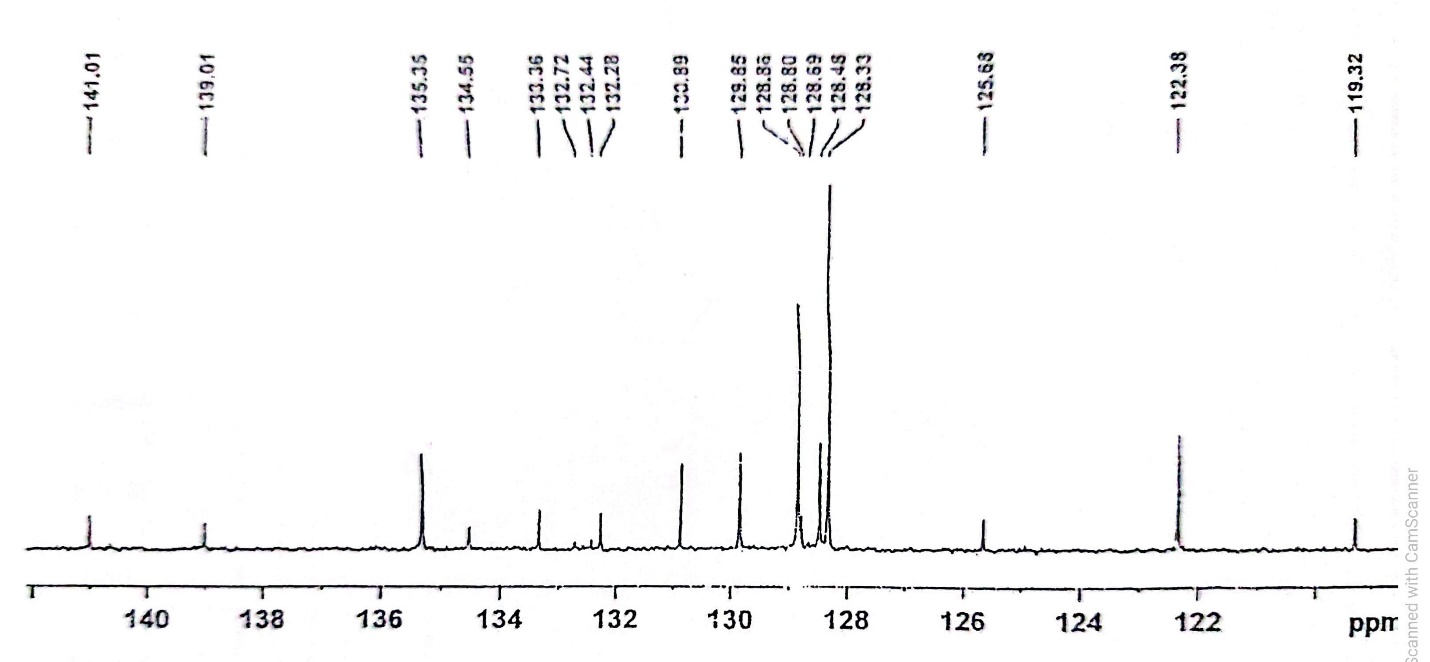


**Figure S14.** Mass spectrum of 2-(4-chlorophenyl)-3-cyclohexylamino-imidazo[1,2-*c*]quinazoline **(4a)**

**
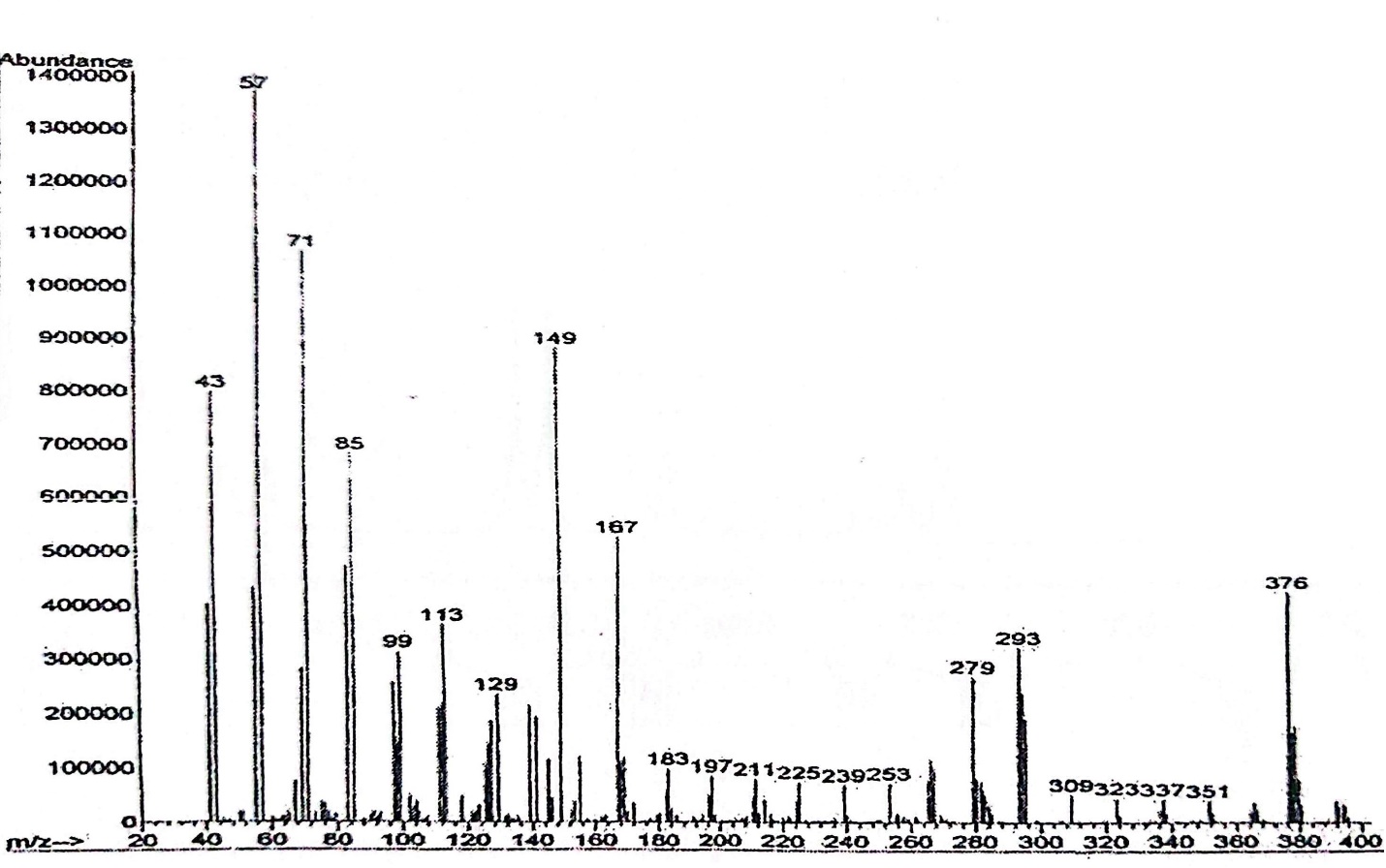
**

**Figure S15.** ^1^H NMR spectrum of 2-(4-fluorophenyl)-3-cyclohexylamino-imidazo[1,2-*c*]quinazoline **(4b)**

**
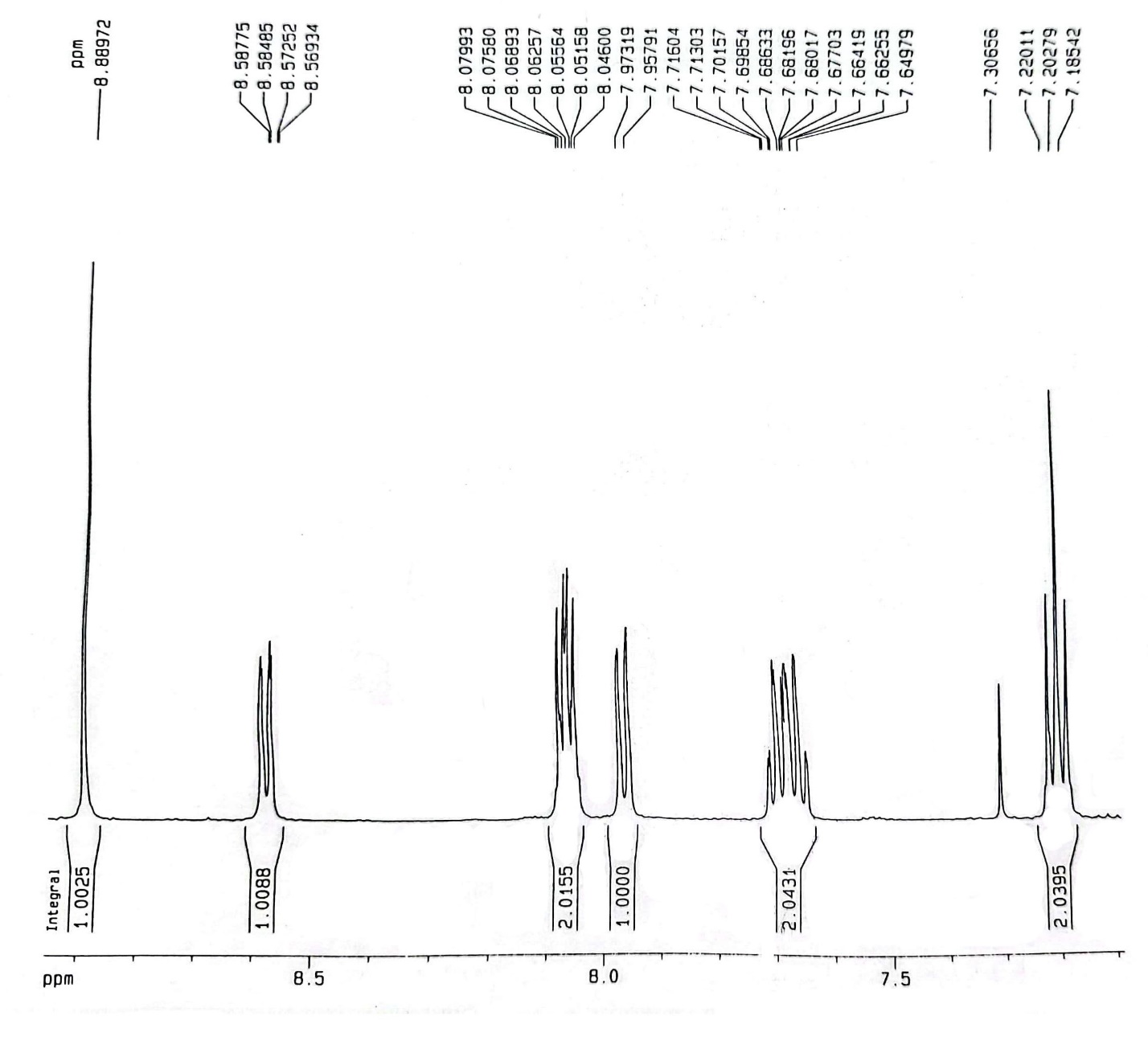

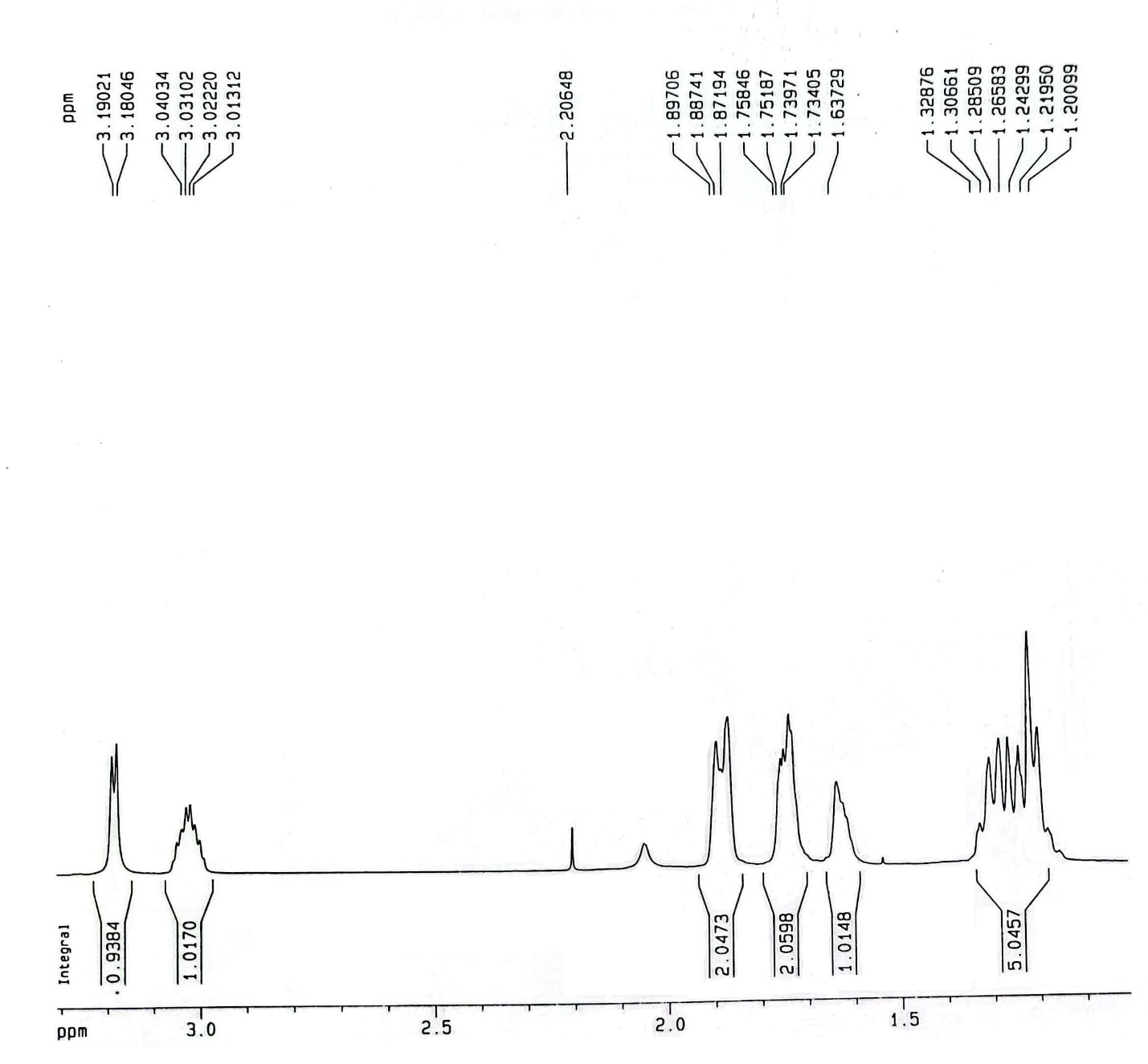
**

**Figure S16.** ^13^C NMR spectrum of 2-(4-fluorophenyl)-3-cyclohexylamino-imidazo[1,2-*c*]quinazoline **(4b)**

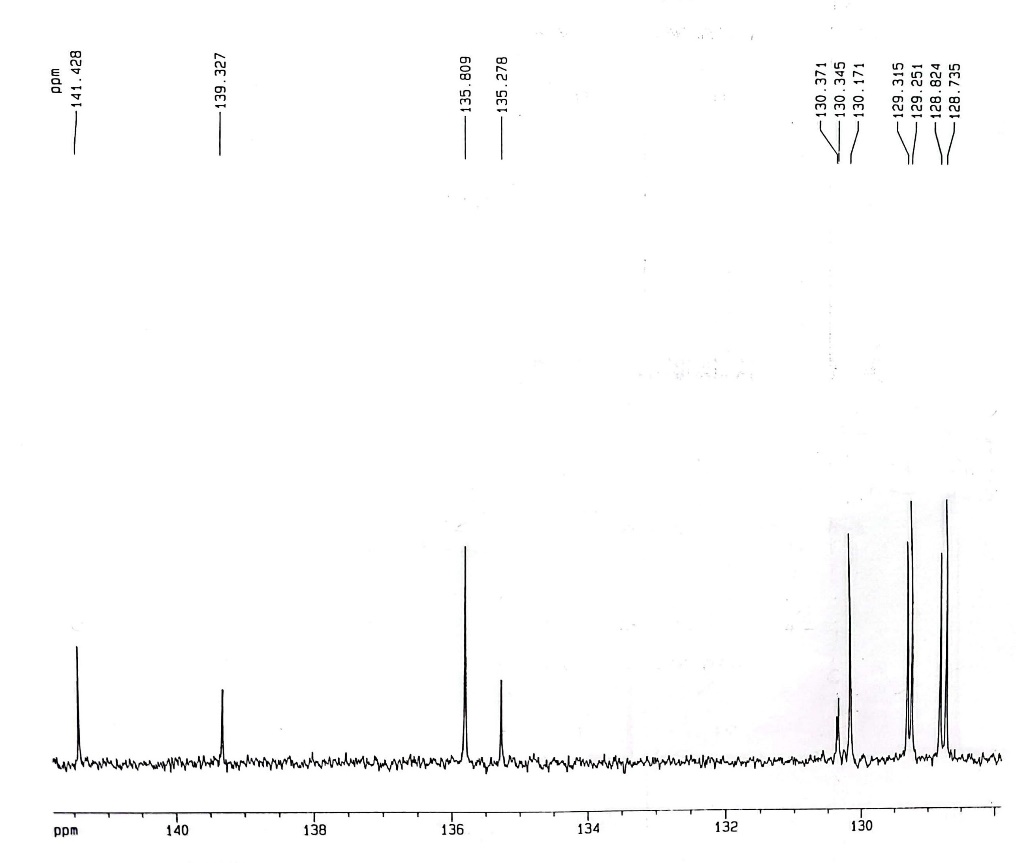


**Figure S17.** Mass spectrum of 2-(4-fluorophenyl)-3-cyclohexylamino-imidazo[1,2-*c*]quinazoline **(4b)**

**Figure S18.** ^1^H NMR spectrum of 2-(4-methoxyphenyl)-3-cyclohexylamino-imidazo[1,2-*c*]quinazoline **(4c)**


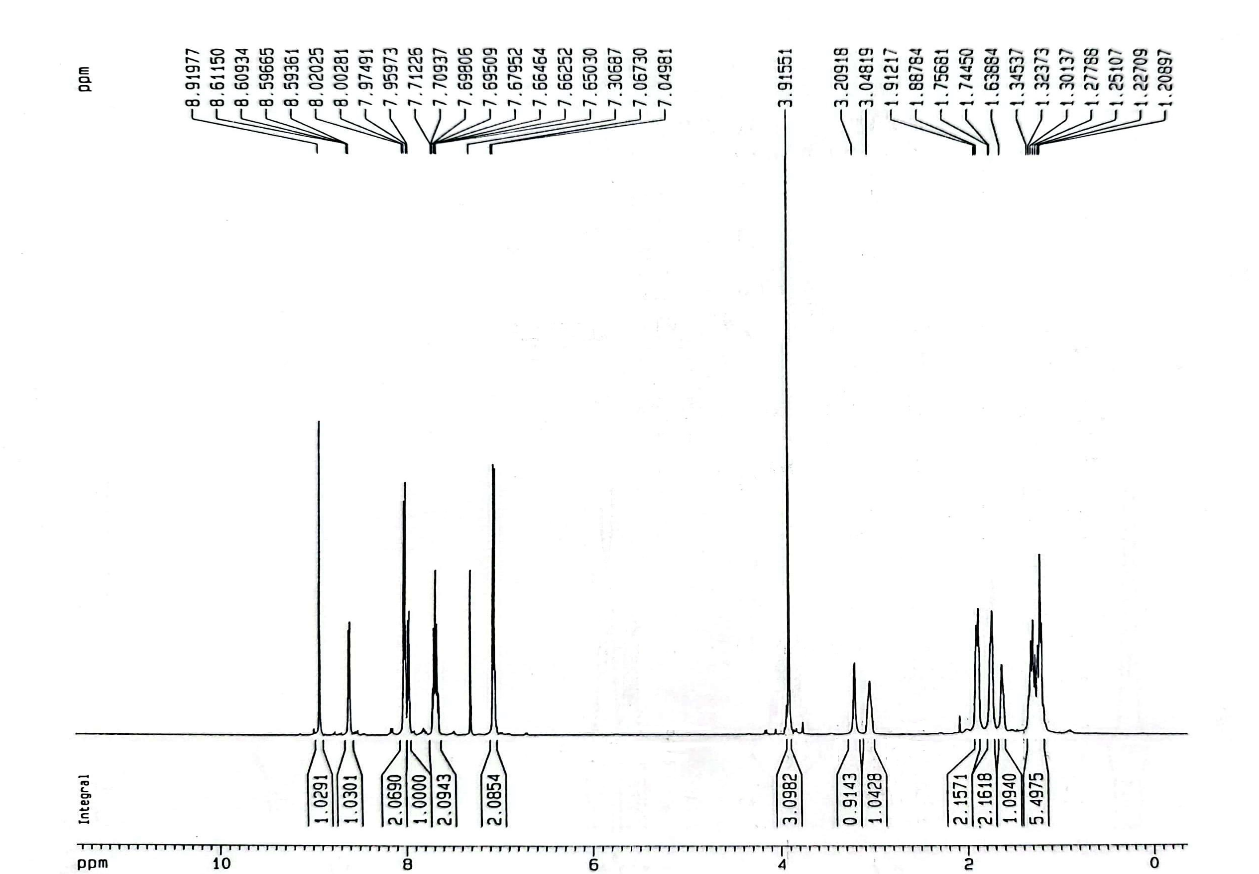


**Figure S19.** ^13^C NMR spectrum of 2-(4-methoxyphenyl)-3-cyclohexylamino-imidazo[1,2-*c*]quinazoline **(4c)**


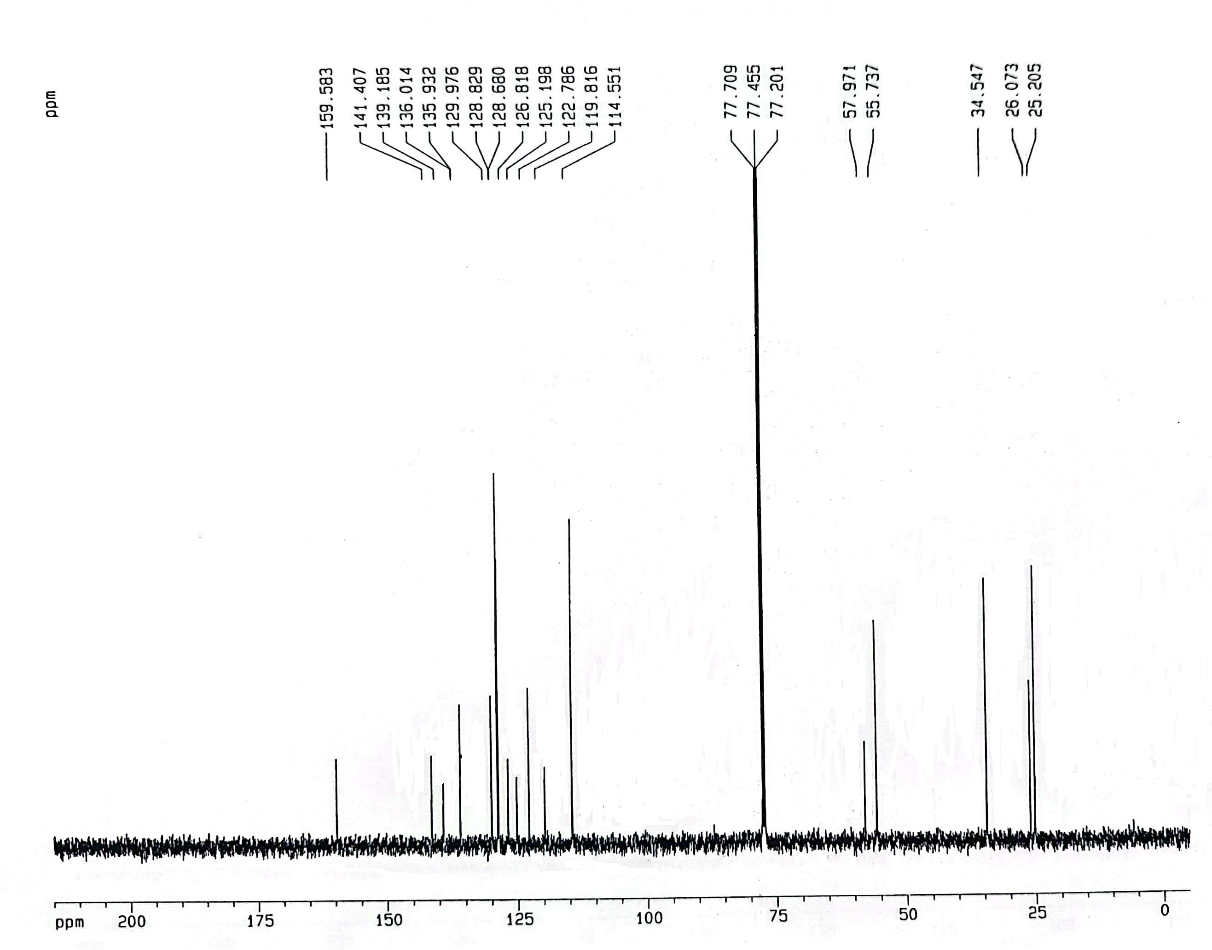


**Figure S20.** Mass spectrum of 2-(4-methoxyphenyl)-3-cyclohexylamino-imidazo[1,2-*c*]quinazoline **(4c)**

**Figure S21.** ^1^H NMR spectrum of 2-(3-chlorophenyl)-3-cyclohexylamino-imidazo[1,2-*c*]quinazoline **(4d)**


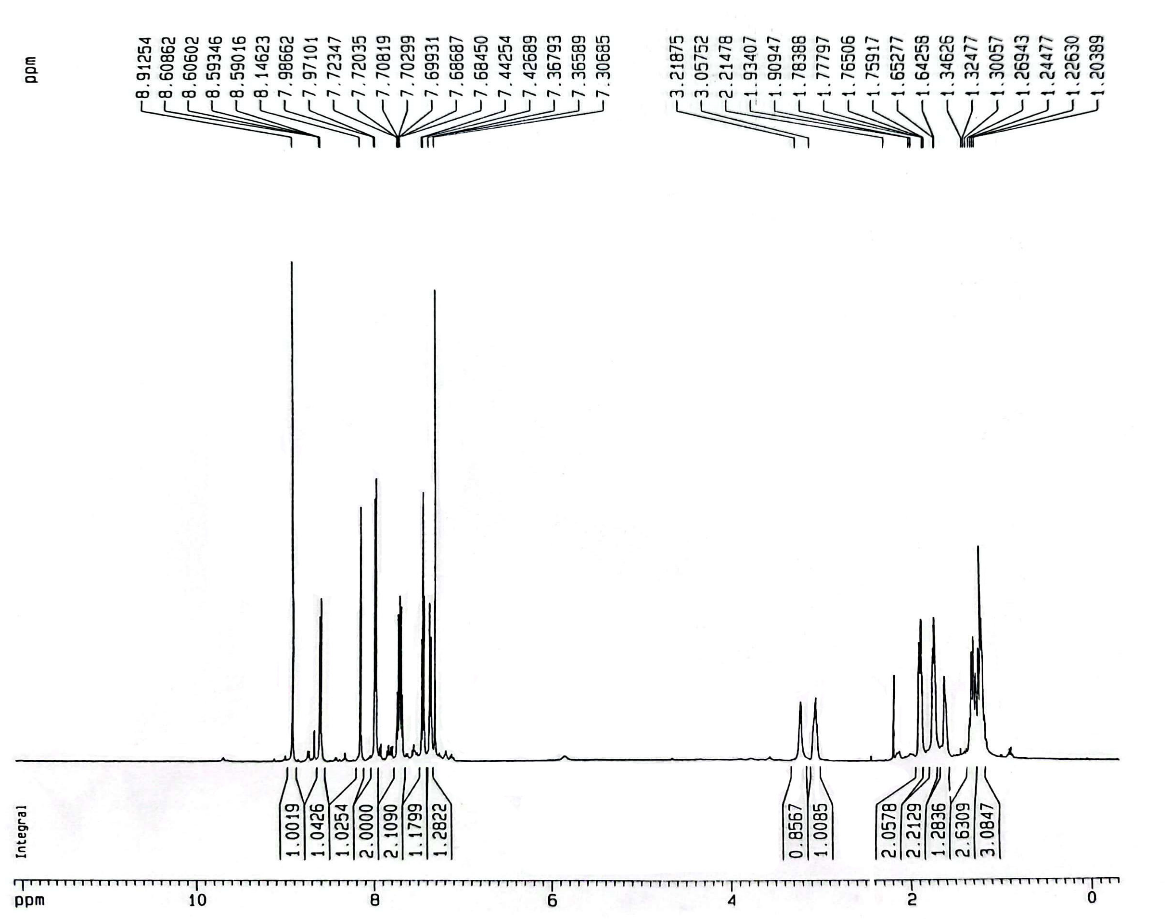


**Figure S22.** ^13^C NMR spectrum of 2-(3-chlorophenyl)-3-cyclohexylamino-imidazo[1,2-*c*]quinazoline **(4d)**


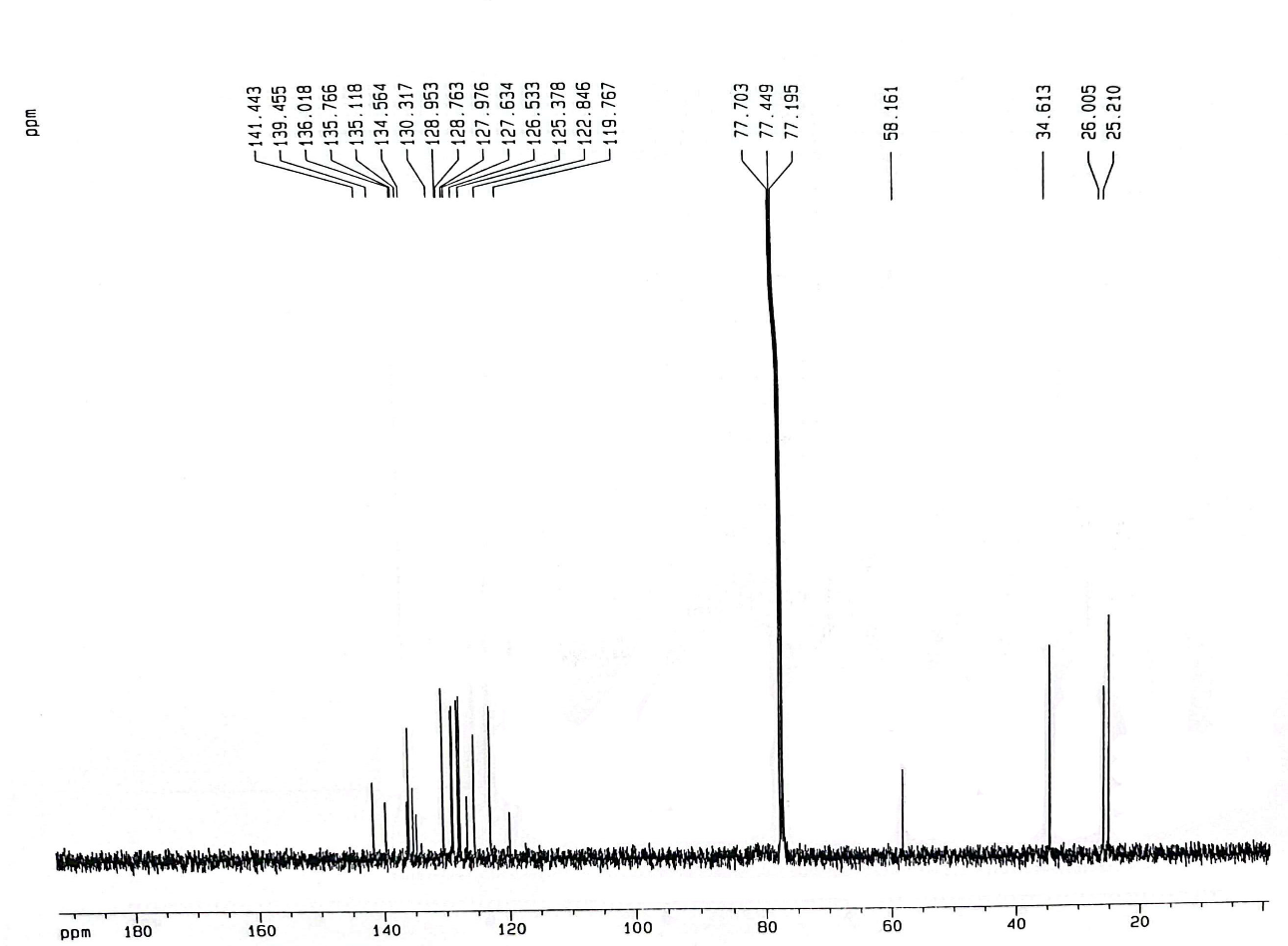


**Figure S23.** Mass spectrum of 2-(3-chlorophenyl)-3-cyclohexylamino-imidazo[1,2-*c*]quinazoline **(4d)**


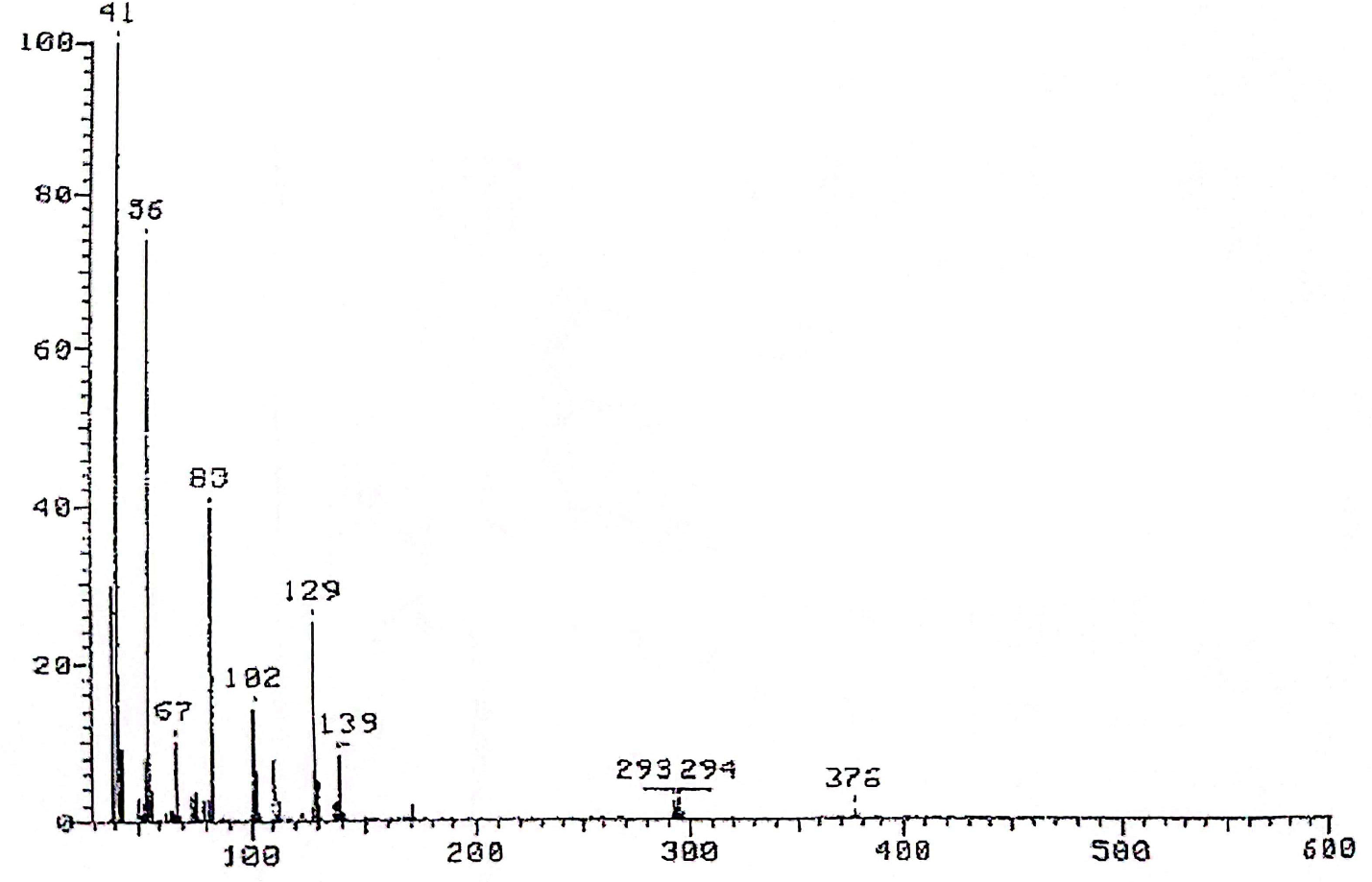


**Figure S24.** ^1^H NMR spectrum of 2-(4-isopropylphenyl)-3-cyclohexylamino-5-methyl-imidazo[1,2-*c*]quinazoline **(4e)**

**Figure S25.** ^13^C NMR spectrum of 2-(4-isopropylphenyl)-3-cyclohexylamino-5-methyl-imidazo[1,2-*c*]quinazoline **(4e)**

**Figure S26.** Mass spectrum of 2-(4-isopropylphenyl)-3-cyclohexylamino-5-methyl-imidazo[1,2-*c*]quinazoline **(4e)**

**Figure S27.** ^1^H NMR spectrum of 2-(4-isopropylphenyl)-3-cyclohexylamino-imidazo[1,2-*c*]quinazoline **(4f)**


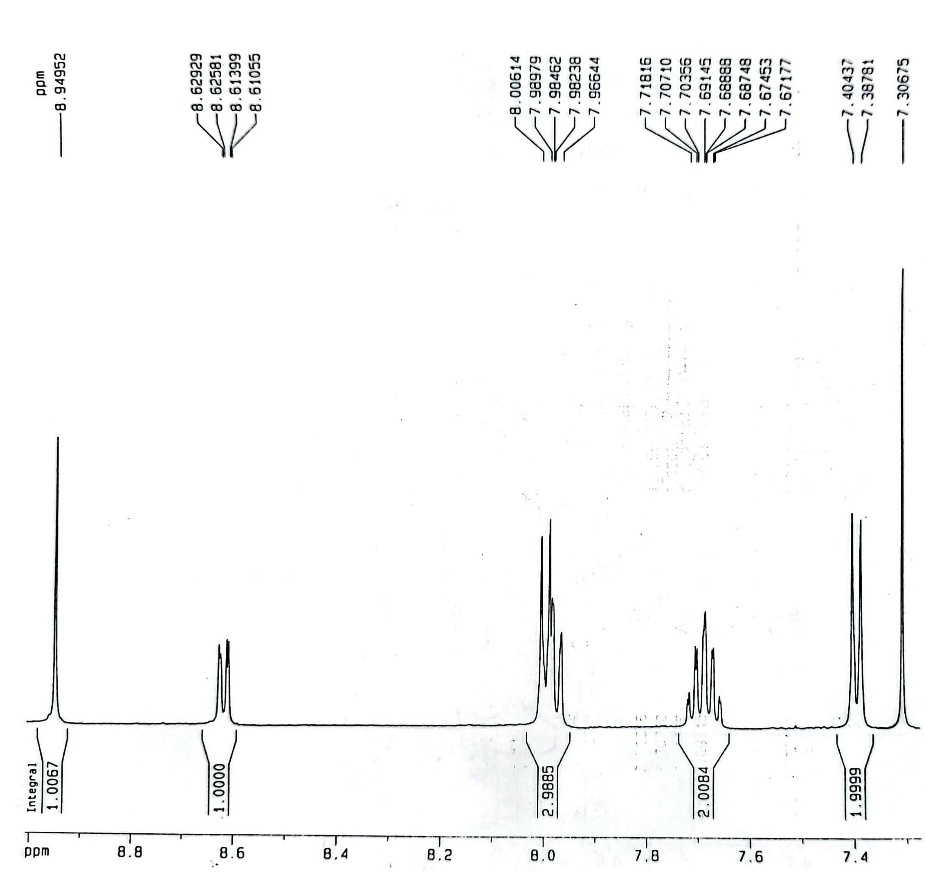


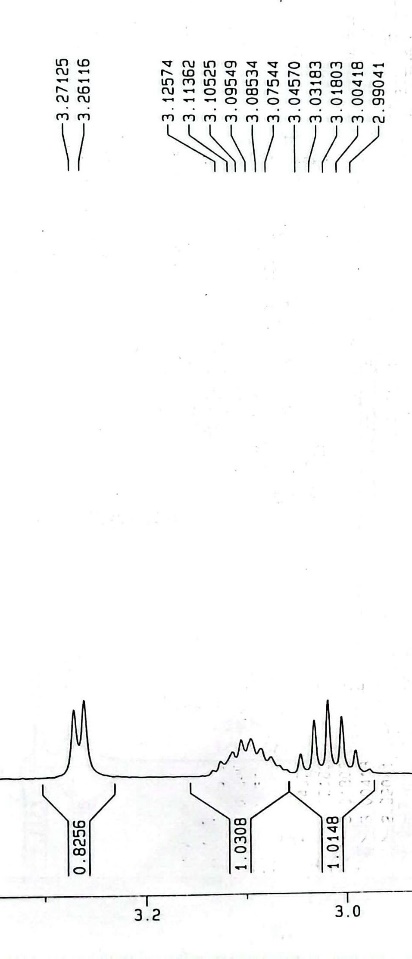


**Figure S28.** ^13^C NMR spectrum of 2-(4-isopropylphenyl)-3-cyclohexylamino-imidazo[1,2-*c*]quinazoline **(4f)**

**Figure S29.** Mass spectrum of 2-(4-isopropylphenyl)-3-cyclohexylamino-imidazo[1,2-*c*]quinazoline **(4f)**


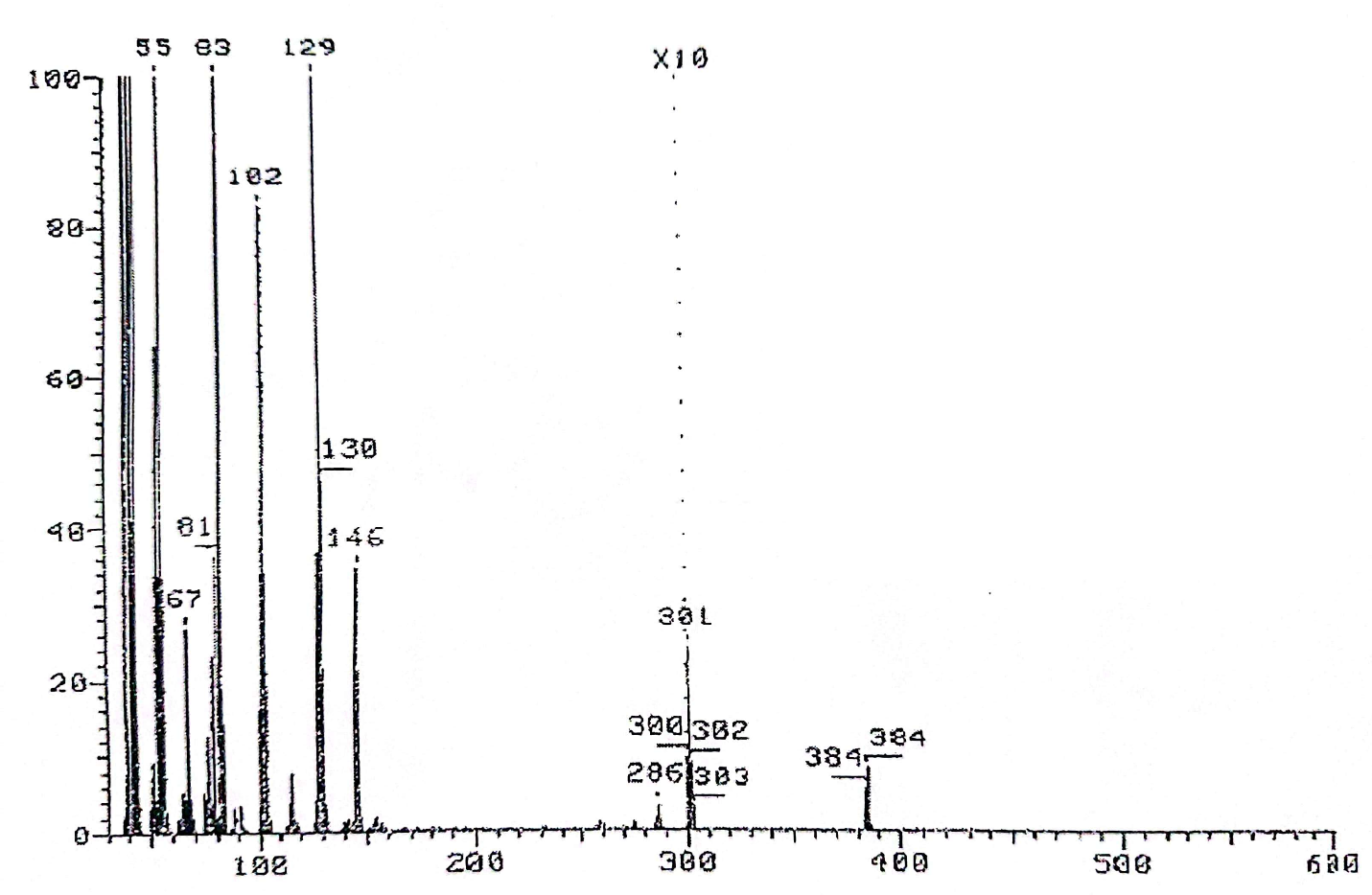


**Figure S30.** ^1^H NMR spectrum of 2-(2,4-dichlorophenyl)-3-cyclohexylamino-imidazo[1,2-*c*]quinazoline **(4g)**


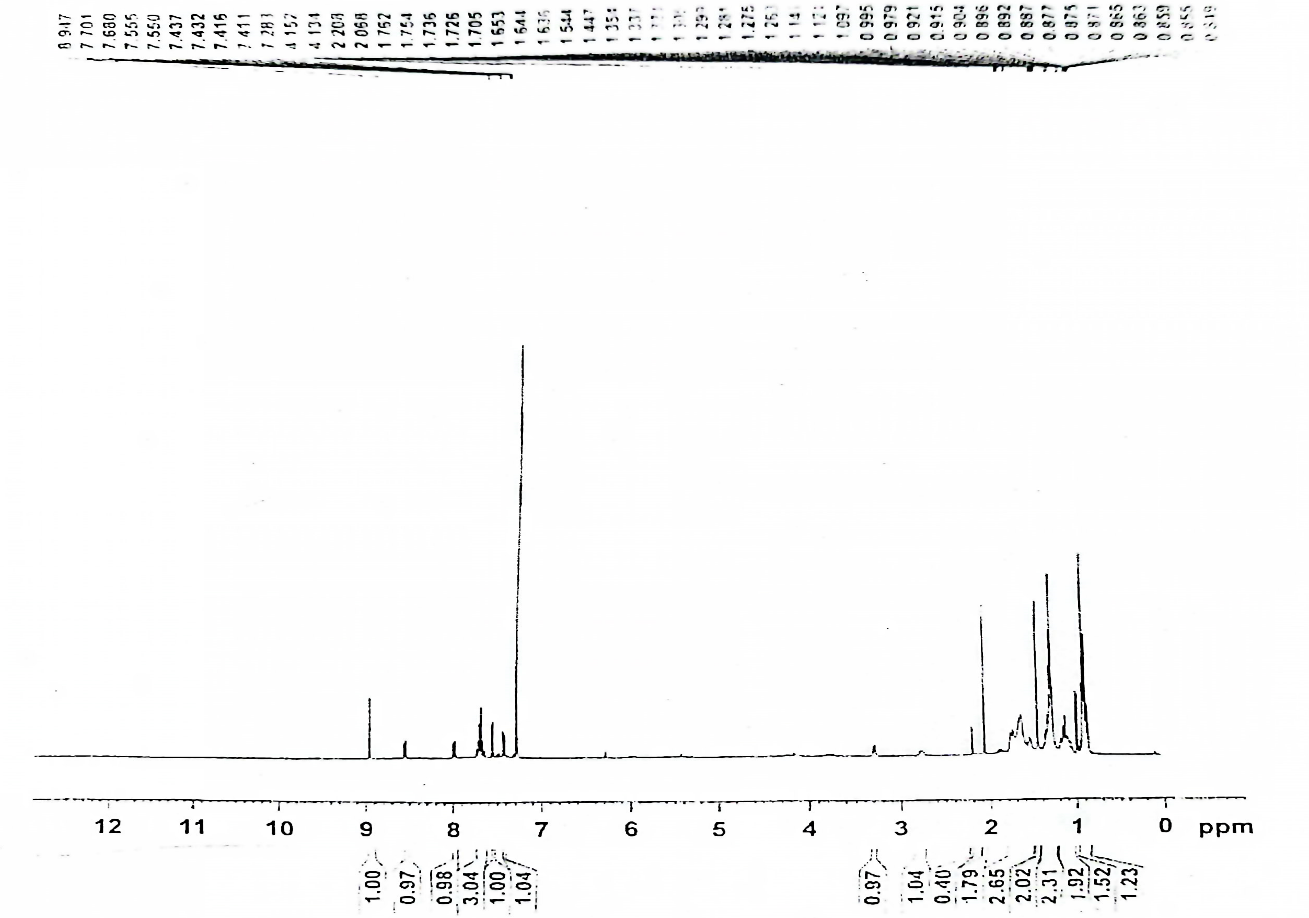


**Figure S31.** ^13^C NMR spectrum of 2-(2,4-dichlorophenyl)-3-cyclohexylamino-imidazo[1,2-*c*]quinazoline **(4g)**

**Figure S32.** Mass spectrum of 2-(2,4-dichlorophenyl)-3-cyclohexylamino-imidazo[1,2-*c*]quinazoline **(4g)**


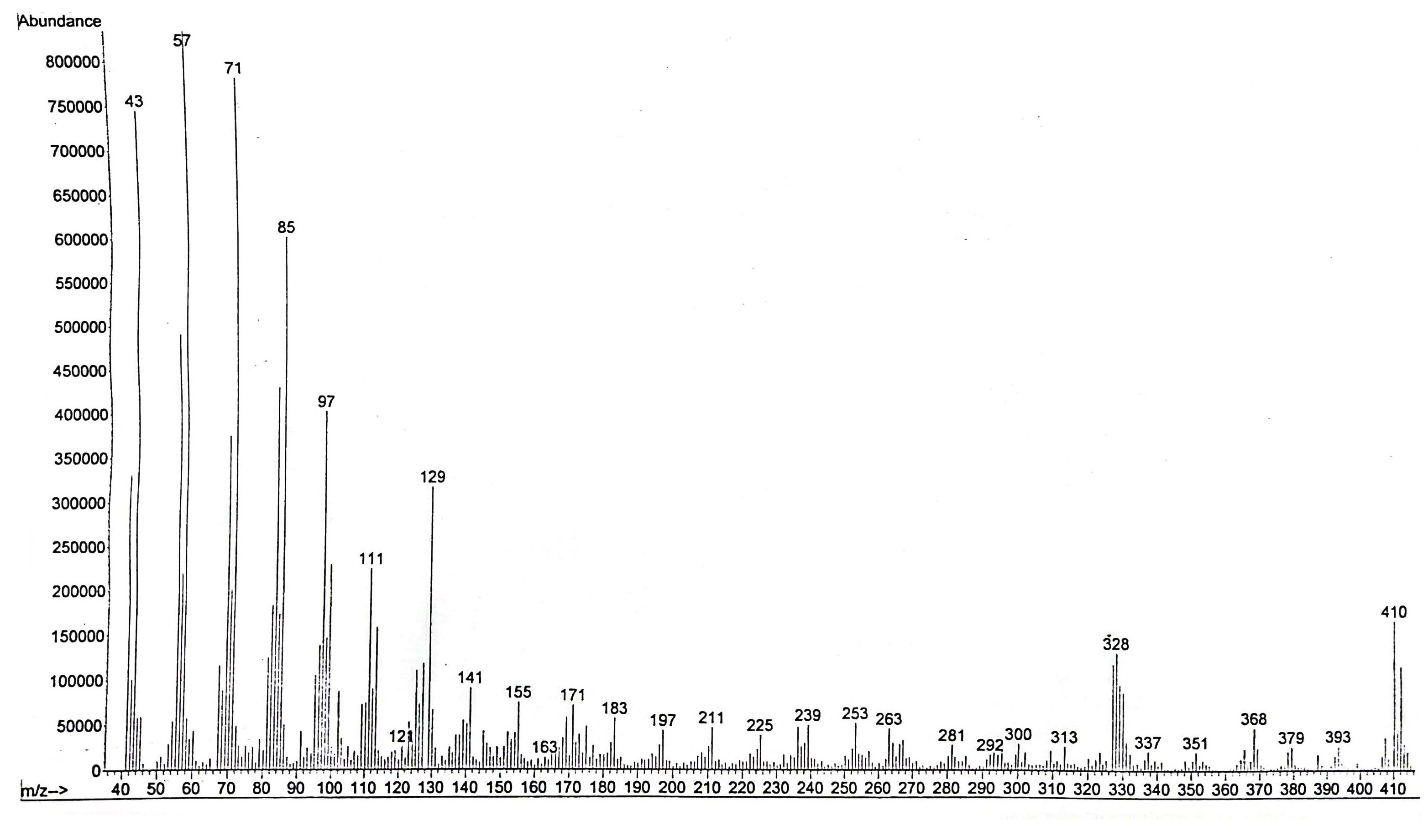

Supplement: Supplementary file 1 — Supplementary Figures. [file 41598_2023_51123_MOESM1_ESM.docx]
